# Bio-Guided Isolation of Acetogenins from *Annona cherimola* Deciduous Leaves: Production of Nanocarriers to Boost the Bioavailability Properties

**DOI:** 10.3390/molecules25204861

**Published:** 2020-10-21

**Authors:** Maria Teresa Gutiérrez, Alexandra G. Durán, Francisco J. R. Mejías, José M. G. Molinillo, Diego Megias, Manuel M. Valdivia, Francisco A. Macías

**Affiliations:** 1 Allelopathy Group, Department of Organic Chemistry, Institute of Biomolecules (INBIO), Campus de Excelencia Internacional (ceiA3), School of Science, University of Cadiz, 11510 Puerto Real, Spain; mariateresa.gutierrezva@alum.uca.es (M.T.G.); alexandra.garcia@uca.es (A.G.D.); javi.rodriguezmejias@uca.es (F.J.R.M.); chema.gonzalez@uca.es (J.M.G.M.); 2 Confocal Microscopy Unit, Spanish National Cancer Research Center (CNIO), E-28029 Madrid, Spain; dmegias@cnio.es; 3 Department of Biomedicine, Biotechnology and Public Health, School of Science, Institute of Biomolecules (INBIO), University of Cadiz, 11510 Puerto Real, Spain; manuel.valdivia@uca.es

**Keywords:** *Annona cherimola*, acetogenins, by-products, encapsulation, cytotoxic activity, nutraceutical

## Abstract

Annonaceous acetogenins (ACGs) are lipophilic polyketides isolated exclusively from Annonaceae. They are considered to be amongst the most potent antitumor compounds. Nevertheless, their applications are limited by their poor solubility. The isolation of ACGs from *Annona cherimola* leaves, an agricultural waste, has not been reported to date. Molvizarin (**1**) cherimolin-1 (**2**), motrilin (**3**), annonacin (**4**) and annonisin (**5**) are isolated for the first time from *A. cherimola* deciduous leaves. Annonacin was found to be four- and two-times more potent in tumoral cells (HeLa, 23.6% live cells; IGROV-1, 40.8% live cells for 24 h) than in HEK-293 at 50 µM (24 h, 87.2% live cells). Supramolecular polymer micelles (SMPMs) were synthesized to encapsulate the major ACG isolated, annonacin, in order to improve its solubility in aqueous media. The bioavailability of this compound was increased by a factor of 13 in a simulated human digestive system when compared with free annonacin and an encapsulation efficiency of 35% was achieved. In addition, the cytotoxic activity of SMPMs that hosted annonacin (100 µM, 24 h, 5.8% live cells) was increased compared with free annonacin in water (100 µM, 24 h, 92% live cells). These results highlight the use of by-products of *A. cherimola*, and their pure compounds, as a promising source of anticancer agents. The use of SMPMs as nanocarriers of ACGs could be an alternative for their application in food field as nutraceutical to enhance the administration and efficacy.

## 1. Introduction

Annonaceous acetogenins (ACGs) are fatty-acid derived natural products found only in species of the Annonaceae family [1]. The main economic interest in this family is focused on the edible fruits of some species, which have attractive organoleptic characteristics. More than 400 ACGs—obtained mainly from roots, stems, seeds and, less often, from leaves [2]—have been described in the last few decades. These compounds contain a long aliphatic fatty acid chain with 35–37 carbon atoms, derived from the polyketide pathway [3], a terminal methyl-substituted α,β-unsaturated γ-lactone ring and either none, one, two or three tetrahydrofuran (THF) rings that can be adjacent or not adjacent to one another [4]. Furthermore, several hydroxyl groups are present in different positions, frequently alongside the THF rings. Overall, the molecular structure comprises two functional moieties: the polar core and the terminal lactone. The biological activity is influenced by the number and stereochemistry of the THF rings and the number of carbon atoms between the polar core and the γ-unsaturated lactone [5]. For instance, it is considered optimum for activity to have at least 13 carbons in the spacer [6].

Spain is one of the major *Annona cherimola* Mill. producers worldwide and the fruit, cherimoya, has become an important crop due to its sensorial and nutritional properties [7]. This species is native to Peru and Ecuador and different parts of this plant have been used in folk medicine for centuries, mainly for the treatment of intestinal and skin diseases [8]. For example, leaves preparations have been used to treat digestive and respiratory disorders and the fruit has been employed for the treatment of stomach-ache and pancreatic ulcers [9]. Other applications of acetogenins in food field are the employment of the cherimoya fruit pulp and avocado seed extract as preservative, due to their antioxidant capacity [10,11]. At least 20 ACGs from this species have been isolated from seeds, stems and roots [2]. However, the isolation of ACGs from *A. cherimola* leaves has not been described to date [12]. These exclusively natural compounds from the Annonaceae family have attracted considerable attention in recent years due to the broad range of biological activities described and the characteristic structures. Cytotoxic, pesticidal, antimicrobial and antimalarial activities, amongst others, have been reported [13]. ACGs are currently considered to be amongst the most potent antitumor compounds. The inhibition of complex I (NADH-ubiquinone oxidoreductase) of mitochondrial electron transport systems [14,15,16], inhibition of glucose uptake (they are potent modulators of glucose transporters) [17] and a target of the hypoxia-inducible factor-1 (HIF-1) have been well established as modes of action [18]. Furthermore, the effectiveness of ACGs against multidrug-resistant human mammary adenocarcinoma and lung cancer cell lines has been described, with better results obtained than for conventional treatments [5,19]. In spite of these advantages, this kind of compound has very low water solubility and their use in clinical or dietary supplement applications is therefore limited. In some in vivo experiments, ethanolic solutions have been used as a vehicle and, in other cases, in order to improve this vehicle ACGs have been dispersed in NaCl solution prior to injection [20,21,22,23,24]. The use of organic solvent does not seem to be a good approach for real applications in human studies. Furthermore, the addition of salt could lead to acid-base modification of functional groups of ACGs, such as hydroxyl groups, which would potentially have an impact on side reactions of the bioactive molecule. In this case, the real activity of the original biomolecule would not be observed but those of a different product would [25]. In order to avoid these undesired effects, nanotechnology has increasingly attracted significant attention to enhance the oral administration or food stability [26]. Nanoparticles have emerged as useful tools in medicinal chemistry for real applications, as in case of β-carotene or curcumin, with remarkable results obtained against melanoma cancer cells [27,28].

The work described here concerns the bio-guided isolation of ACGs from *Annona cherimola* Mill. deciduous leaves. The investigation of the potential of this by-product could lead to less waste, potential health benefits and an increase industrial profitability. In vitro experiments on different tumoral (human ovarian (IGROV-1) and cervix carcinoma (HeLa) cell lines) and non-tumoral (human embryonic kidney 293 (HEK-293)) cells were performed to evaluate the cytotoxicity of extracts and pure compounds. A supramolecular polymer micelle was synthesized to encapsulate the main ACG isolated from *A. cherimola* leaves, namely annonacin, in an effort to improve the drug-release system [29]. Bioavailability in a simulated gastrointestinal model, stability and solubility were also investigated.

## 2. Results and Discussion

### 2.1. Bio-Guided Isolation

**Figure 1:**
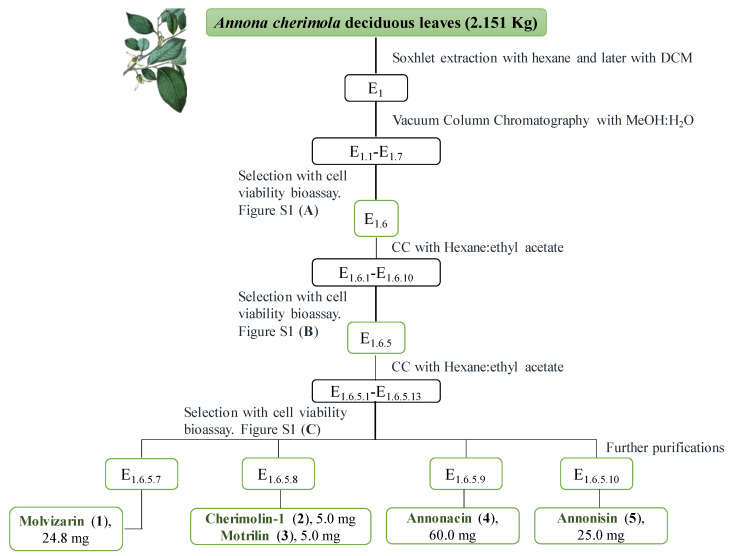
Bio-guided fractionation of *A. cherimola* deciduous leaves. For Figures S1A–C please see supporting information.

The isolation of ACGs from *Annona cherimola* leaves has not been reported to date. It is worth highlighting the use of this by-product (deciduous leaves) as a source of bioactive ACGs. Although the study and purification of this kind of compounds can require multiple separation techniques [2], five ACGs were obtained in this study by bioassay-guided fractionation (Figure 1 and Figure S1).

**Figure 2:**
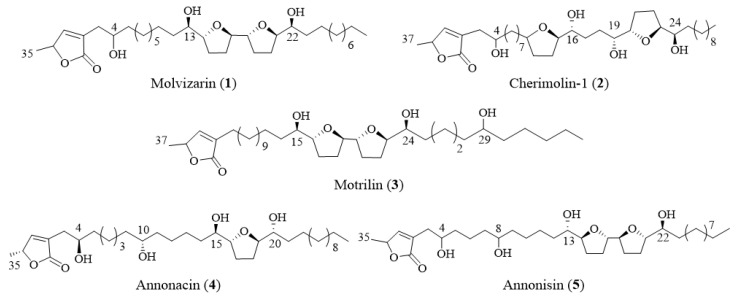
Annonaceous acetogenins (ACGs) isolated from *Annona cherimola* deciduous leaves.

The structures were elucidated based on HRMS and NMR experiments. The spectroscopic data are consistent with those described previously for: three adjacent bis-THF (molvizarin (**1**) (24.8 mg, 0.001%), motrilin (**3**) (5 mg, 0.0002%), and annonisin (**5**) (25 mg, 0.001%)), one mono-THF (annonacin (**4**) (60.0 mg, 0.003%)) and one non-adjacent bis-THF acetogenin (cherimolin-1 (**2**) (5 mg, 0.0002%)) (Figure 2) [30,31,32,33]. For physical data see supporting information (Supplementary Materials Pages S3–S8). These compounds are described for the first time in *Annona cherimola* Mill. deciduous leaves and one of them (annonisin) has never been isolated from this species before. Yields obtained were similar or even higher to those reported for this family of compounds isolated from Annonaceae [34,35,36].

### 2.2. Cytotoxic Activities of Isolated ACGs

**Figure 3:**
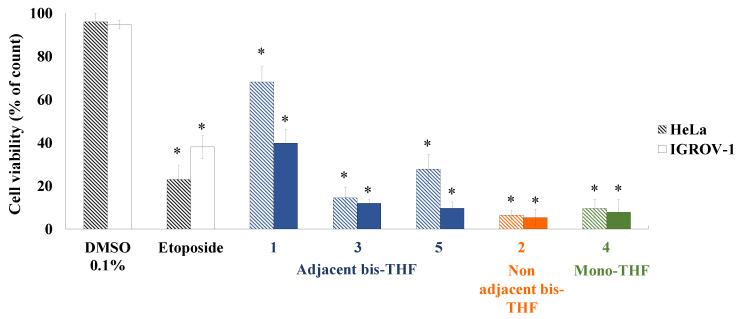
Cell viability by dye exclusion against ovarian (IGROV-1) and cervix carcinoma (HeLa) cells. ACGs **1**–**5**, classified by skeleton using different colors, and positive control (etoposide) were evaluated at 100 µM for 24 h. Experiments were performed in triplicate and data are expressed as mean ± SD, *n* = 3, * *p* < 0.05 vs. untreated cells (DMSO 0.1%).

Isolated ACGs were evaluated against IGROV-1 and HeLa tumoral cell lines at 100 µM for 24 h. (Figure 3). Significant cytotoxic activities were observed for all of the ACGs tested. The highest activity values were obtained for compounds **2** and **4**, which gave cell viability values of less than 10% and were more active than positive control (etoposide). Lower activity was observed when cells were treated with ACG **1**, which was approximately seven- and four-times less active than ACGs **2** and **4** for the Hela and IGROV-1 cell lines. In several studies it has been demonstrated that structural features, such as the number of hydroxyl groups on the aliphatic chain and the spacer between the THF and lactone ring, are crucial factors that influence the activity [37]. In this particular case, it can be suggested that non-adjacent bis-THF (**2**) and mono-THF (**4**) ACGs showed better inhibition than adjacent bis-THF ACGs (**1**, **3** and **5**). Moreover, the isolated ACGs were slightly more potent against IGROV-1 cells at 100 µM for 24 h.

**Figure 4:**
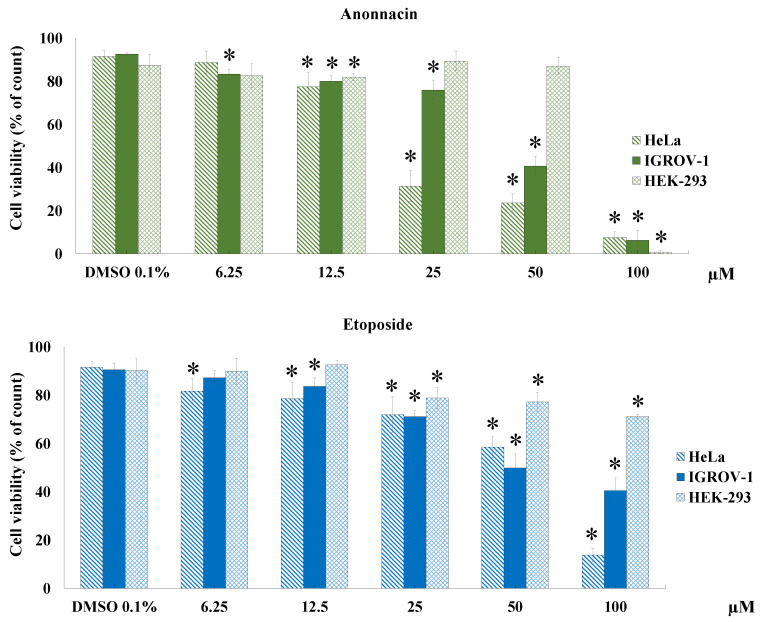
Cell viability by dye exclusion against ovarian (IGROV-1) cervix carcinoma (HeLa) and human embryonic kidney 293 (HEK-293) cells. The ACG and etoposide (positive control) were evaluated at 6.25, 12.5, 25, 50 and 100 µM for 24 h. Experiments were performed in triplicate and data are expressed as mean ± SD, *n* = 3, * *p* < 0.05 vs. untreated cells (DMSO 0.1%).

In view of the results outlined above, one of the most active ACGs that was also isolated in higher amounts, namely annonacin (**4**), was selected to perform a cytotoxicity study at various concentrations (from 6.25 to 100 µM) in different tumoral (IGROV-1 and HeLa) and non-tumoral (HEK-293) cell lines (Figure 4) for 24 h. It is worth nothing that compound **4** showed selectivity as it proved to be approximately four- and two-times more potent in tumoral cells (HeLa and IGROV-1 respectively) than in non-tumoral human embryonic kidney cells (HEK-293) at 50 µM. This variation in cytotoxicity is a useful property in the search for potential anticancer compounds. IC_50_ values of 19.32 (R^2^ = 0.9823), 46.54 (R^2^ = 0.9791) and 68.76 µM (R^2^ = 0.9813) were obtained for HeLa, IGROV-1 and HEK-293 cells, respectively. IC_50_ values after treatment with annonacin were less than positive control etoposide in tumoral cell lines (HeLa 73.67 µM (R^2^ = 0.9942) and IGROV-1 59.89 µM (R^2^ = 0.9607)). Selectivity index, (SI value expressed as IC_50_ ratio in HEK-293 cells versus HeLa/IGROV-1 cells) was calculated so as to evaluate the selectivity of annonacin to the cell lines tested. Compounds are classified as high selective if the SI value is ≥ 3 and less selectivity if it is lower than 3 [38]. Results showed that **4** possess a high degree of cytotoxic selectivity (SI = 3.55) to HeLa cells while it showed less selectivity to IGROV-1 cells (SI = 2.40).

**Figure 5:**
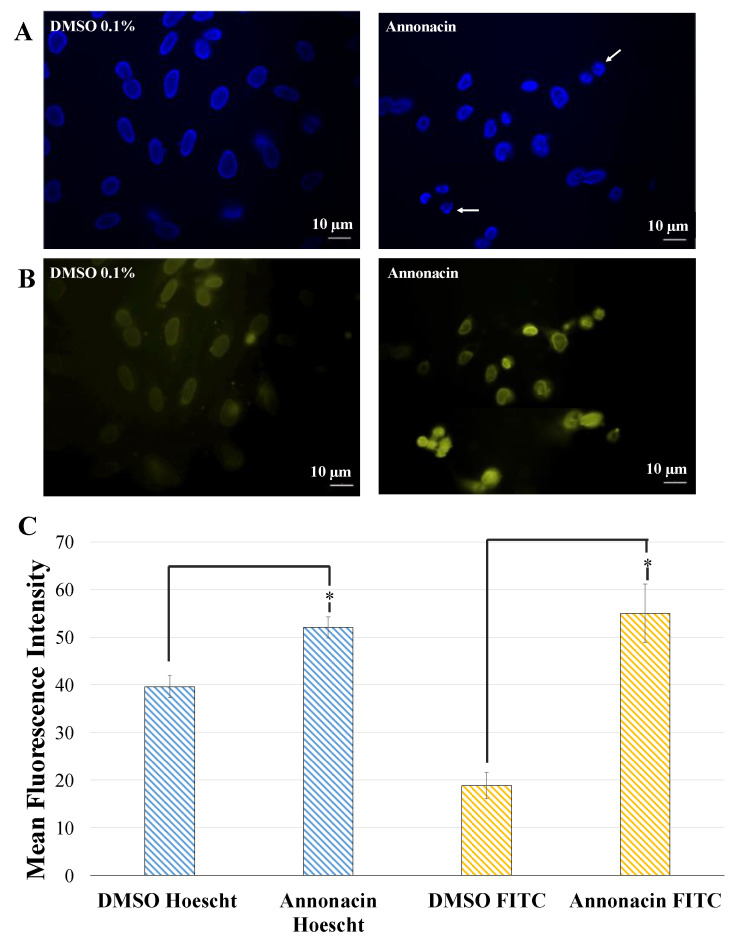
(**A**) DNA fluorescence staining by Hoechst after treatment with DMSO 0.1% and annonacin at 100 µM respectively for 20 h on HeLa cells. (**B**) Secondary anti-rabbit IgG-FITC antibody of HeLa cells treated with DMSO 0.1% and annonacin at 100 µM respectively for 20 h. (**C**) Quantification of mean fluorescence intensity signal in HeLa cells treated with DMSO 0.1% and annonacin after indirect immunofluorescence caspase-3 assay. Data are expressed as mean ± SEM, *n* = 3, * *p* < 0.05 vs. untreated cells (DMSO 0.1%).

Caspases play a key role in the apoptotic signaling pathways and caspase-3 is considered to be the most important of the executioner caspases. After cleavage by caspase-3, DNA fragmentation and a more pronounced and advanced chromatin condensation, or disintegration of the cell into apoptotic bodies take place [39]. To evaluate the mechanisms of annonacin-induced cell death, indirect immunofluorescence caspase-3 study was carried out. Results revealed that the expression intensity of fluorescent caspase-3 on HeLa cells after treatment with annonacin was higher than that in control group (DMSO 0.1%). These results could confirm that annonacin triggered apoptotic cell death on Hela cells which led to nuclei condensation and fragmentation, as it is indicated in its increase in fluoresce-staining (Figure 5). Our observations in the induction of apoptosis in the cell lines studied corroborate previous studies with ACGs [40,41]. It is well documented that the biological activities of ACGs are primarily characterized with toxicity against cancer cells and inhibitory effects against the mitochondrial complex I (mitochondrial NADH: ubiquinone oxidoreductase). That mechanism includes primarily dose-dependent inhibition of complex I of the respiratory chain. Specifically, it has been described that Bis-THF motif of acetogenins binds to the third matrix-side loop of ND1 subunit in mitochondrial NADH-ubiquinone oxidoreductase. The differences observed between the different tumors lines can be explained hypothetically among other reasons by the contribution, in addition to the induction of apoptosis, of additional mechanisms of action such as the influence on glucose transporters and that of hypoxia-inducible factor-1. In this regard, studies have observed that certain ACGs lead to accumulation of intracellular reactive oxygen species (ROS) at the early stage and they may trigger the cell death through a caspase-3 independent pathway. This in turn may explain also the results observed in our case in the use of the non-tumor line HEK-293 where they have been different from those of the tumor lines and may contribute in some way to explain the variability of results observed.

### 2.3. Synthesis of SMPMs of Annonacin

**Figure 6:**
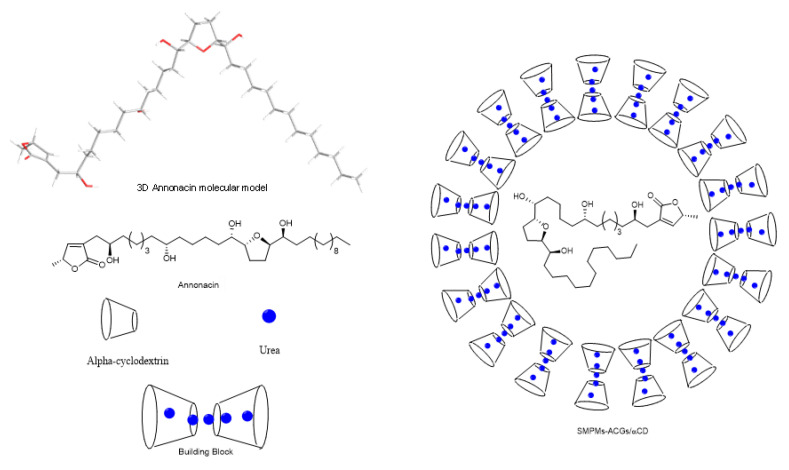
3D model of annonacin and the formation of supramolecular polymer micelles.

A supramolecular polymer micelle was synthesized to encapsulate the annonacin. The building blocks of the system were α-cyclodextrin and urea (Figure 6). These compounds form the hydrophilic part of a micelle, with the aim of increasing the solubility and bioavailability of annonacin. Intermolecular forces, such as hydrogen bonds, π-π-stacking interactions, electrostatic and van der Waals forces [42,43], established between the polyrotaxane and acetogenins allow the annonacin to be locked within the shell formed by the cyclodextrin with urea inside the toroid structure. Vigorous mixing between the water phase, which contained the cyclodextrin and urea, and the organic phase, which contained the annonacin, led to the ‘sequestration’ of the acetogenin into the α-CD/urea inclusion complex. The synthesis procedure required a temperature of 40 °C or above to favor micelle formation. However, the temperature was kept at 40 °C to avoid annonacin degradation (<60 °C).

Under the above conditions ~35% ACG encapsulation was achieved, as determined using the UV-Vis calibration curve obtained at 284.9 nm. The structural integrity of annonacin was verified by ^1^H-NMR spectroscopy on the supramolecular polymer micelles (SMPMs) after release of the encapsulated acetogenin with CDCl_3_. Critical micellar concentration was afforded to analyze the correct formation of the micelles. A CMC experiment has been carried out following the method previously described in the literature [44,45,46]. The SMPMs structures present a non-polar cavity where β-carotene can be hosted offering a less energetic state. Initially, the carotenoid is fully insoluble in water media, but the formation of the micelle allows to increase the solubility and it will decrease the absorbance due to entrapment inside the micelle. According to this idea, 14 samples with the same amount of β-carotene (1 mg) were prepared and the concentration of α-CD and urea were increased gradually. Figure S8 shows a progressive ascending with the concentration of the encapsulation agents which meet to a co-dissolution of all the compounds. Nevertheless, at 10 mM appears a sharp decrease of the absorbance which point out the formation of the micelles and the input of the β−carotene.

### 2.4. Characterization of SMPMs by NMR Spectroscopy and Electron Microscopy

The influence of urea encapsulation and SMPM formation could be evaluated by the ^1^H-NMR shifts of α-cyclodextrin (Figure S2). When urea molecules are hosted by cyclodextrins, the H_1_ signal is shifted most strongly to low field. Furthermore, the H_4_ signal is also shifted to low field. These changes suggest that the most relevant interaction between the cyclodextrin and the urea involves the ether oxygen of an α-1,4 bond through dipolar interactions. In the case of supramolecular micelle formation, all of the proton signals had modified positions and the resolution decreased. This change is observed because the micelles are not in a true solution—a situation that reduces the solvent interaction and makes the spectra more similar to solid NMR spectra. The authors suggest that the –OH groups are arranged outside the micelles, thus displaying a hydrophilic face, which would imply a change in the most stable conformation (chair) and would explain the marked changes in the shifts of all proton signals. Finally, the micelles are fully broken upon treatment with an organic solvent, such as phenol, and the original signals were restored.

The results of ROESYAD 2D experiments (Figure S3) were not conclusive due to the low resolution of the annonacin signals. The inclusion of annonacin within the SMPMs hinders the elucidation and the system behaves as a quasi-solid NMR sample, as shown by the spectrum of α-CD in Figure S2c. Evidence for correlation between signals was not observed for annonacin and cyclodextrin in the ROESYAD 2D experiments and this leads to two possible conclusions. Firstly, it is possible that the limitations of the experiment preclude the elucidation of a spatial relationship between shell and core in SMPMs. The second explanation is a possible inter-arrangement of urea units that establishes a ‘bridge’ of intermolecular forces between the acetogenin and the α-CD. In this case, the ^1^H signals of urea were not observed because of rapid interchange of the amine hydrogen atoms with the solvent.

**Figure 7:**
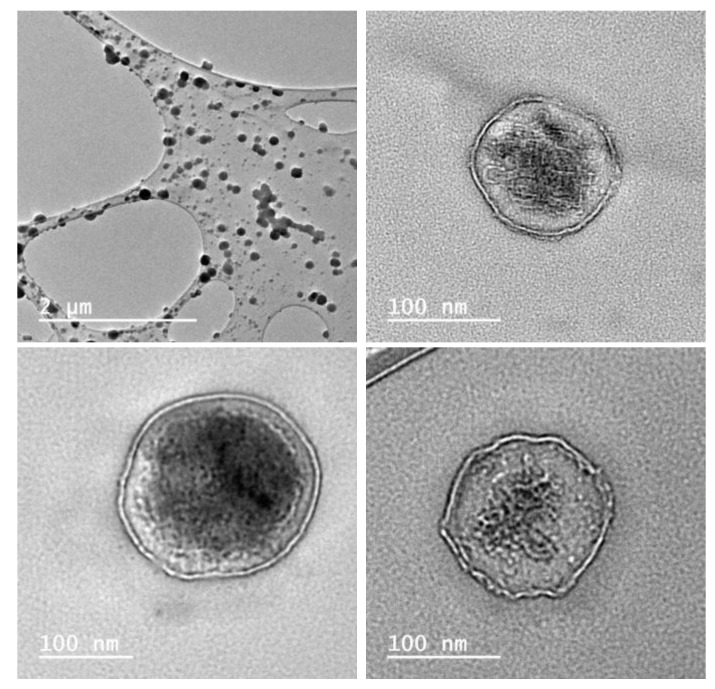
TEM images of supramolecular polymer micelles (SMPMs)-ACGs.

The morphologies of the SMPMs were studied by transmission electron microscopy. The use of a staining process with heavy elements was avoided because the presence of contaminant compounds or agglomeration could hinder the analyses by microscopy. The images (Figure 7) are consistent with the structure proposed in Figure 6, where the polyrotaxanes composed of α-CD with encapsulated urea form the hydrophilic phase of the micelles. The morphologies are predominantly spherical with a wall thickness of 8 ± 1 nm (Figure S4). There seem to be two main groups of SMPMs present, one with sizes between 100 and 160 nm in diameter and the other with diameters between 80 and 30 nm (Figure S5). It is worth mentioning that despite the large sizes of these particles they are still within the nanomedicine application range to overcome the barriers of liver and spleen, according to Cabral and co-workers [47]. According to the literature [48], the shell sizes are around 6 nm and it was concluded that this is consistent with two α-CD units linked by secondary intermolecular forces with several units of urea. Nevertheless, two semi-empirical (PM3) simulation studies with two units of the cyclodextrin and thirty two units of urea (1:16 molar ratio) (Figure S6) were carried out. By approximating the model proposed by Dong et al., a truncated cone geometry between two cyclodextrins was found, with urea molecules filling the outer- and inter-spaces where the narrow parts of the cone are closer than the wider parts. This simulation gave a shell thickness of ~18 Å. Given the significant difference compared to the thickness values reported in the literature, a biased geometry between cyclodextrins was assessed. In the biased case a minimum conformation energy was obtained for an α-CD distance of ~26 Å. The authors therefore suggest that between six and eight α-cyclodextrin molecules connected with urea units constitute the membranes in this specific SMPM with annonacin. The limitations in the theoretical calculation method preclude an explanation for the particular stability of the 8 mm shell for the encapsulated ACG, which is a common feature of all of the TEM images.

Focusing on the larger SMPMs, the internal volume of the micelle is in the range between 3.10 × 10^−4^ and 1.56 × 10^−3^ µm^3^. The molecular volume of the annonacin was calculated by the semi-empirical PM3 method and a molecular volume of 1.715 × 10^−9^ µm^3^ was obtained, which in turn means a range of 0.3–1.514 attomole of annonacin per SMPM in the case where the micelle has a diameter of over 100 nm.

X-ray diffraction from 3.5 to 75.0 2θ degrees were afforded to corroborate that structures observed are micelles and they are not crystallized cyclodextrins, or any crystal derivatives. According to Figure S9, it can be seen that SMPMs are completely amorphous, in comparison with crystallinity showed by α-cyclodextrin sample. If the SMPMs-ACGs sample were composed by cyclodextrins crystallites or crystal-like structures, peaks could be observed in X-Ray diffraction experiment, instead of broad signals that are observed in Figure S9B.

### 2.5. Controlled Release Study

**Figure 8:**
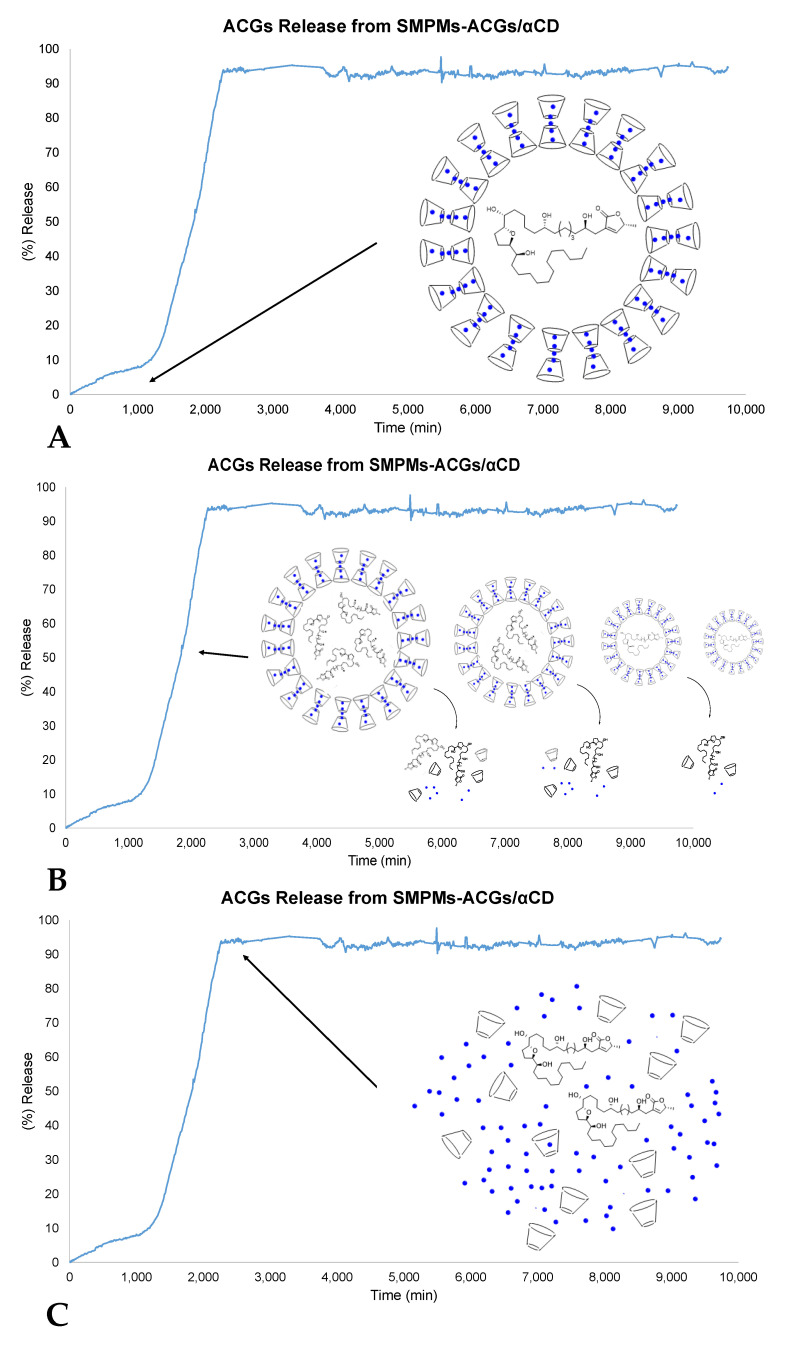
Delivery of annonacin from SMPMs according to UV-Vis studies. (**A**) Early stages with stable SMPMs. (**B**) Delivery process according to the Hixson–Crowell model. (**C**) Stationary state with bioavailable annonacin.

Release profile analysis become necessary to understand future in vivo studies. The time required to release completely to solution acetogenins from the SMPMs shell was studied at 37 °C in saline buffer at pH 7.4. These experiments provided information on the time period over which annonacin is stable in putative biological media such as blood. As a first approach, the variation in UV-Vis intensity at the characteristic wavelength for annonacin (284.9 nm) was followed to evaluate the steps involved in the delivery process. In the first 17 h, α-cyclodextrin and urea maintained a stable shell structure, with a time of 33 h passing until the release process was triggered. The delivery profile shown in Figure 8 indicates that the encapsulated compound is available in PBS solution for at least seven days. However, the behavior in cell media could be slightly different due to the crossing of lipophilic membranes or the action of enzymes.

Different kinetic models were tested to explain the drug release behavior of the annonacin in the medium investigated (Zero order, First order, Higuchi Model, Korsmeyer–Peppas Model, Weibull Model and Hixson–Crowell Model) [49,50]. Two models came close to fitting the behavior of the SMPMs, namely the first order and Hixson–Crowell models, with the latter being the most accurate:

W_t_^1/3^ = W_0_^1/3^ − k·t   R^2^ = 0.959 (Hixson–Crowell model)

Ln (W_t_) = Ln (W_0_) − k·t   R^2^ = 0.885 (First order model)

The Hixson–Crowell method indicates that SMPMs have a regular area that is proportional to the cube root of their volume. W_0_ is the initial amount of annonacin, W_t_ is the remaining amount of annonacin encapsulated at time *t*, and k is a constant that reflects the surface/volume relation. The expression represents the release from a system in which there is a change in the surface area and diameter of particles. Equilibrium conditions are not modified and the surface of the SMPMs decreases proportionally over time, in such a way that the initial geometrical remains constant (Figure 8B) [51]. In this model it is assumed that the annonacin release is limited by dissolution velocity and not by diffusion [52]. In this particular case, the experimental data can be expressed as W_t_^1/3^ = −0.2073 − 0.0016·t.

A comparative study on the bioavailability of free annonacin and encapsulated annonacin showed an improvement by a factor of 13 on using SMPMs with α-CD and urea. The hydrophilic surface provided by the shell part of cyclodextrin and urea allowed the transport and dissolution of the active compound in a simulated oral phase. The majority of the ACGs are then degraded by the strongly acidic medium present in the gastric phase, but the supramolecular structure retains enough protected annonacin to transport it to the intestinal phase. The neutral medium in this latter phase does not seem to damage the micelles, but high concentrations of enzymes, such as lipase, could contribute to the hydrolysis of cyclodextrins. Free annonacin showed a value of 3.68%, but most of the sample could not be solubilized in the initial oral phase due to its low water solubility. Encapsulated annonacin showed a bioavailability of 47.01%, which supports the use of SMPMs in aqueous media over free ACGs.

### 2.6. Cytotoxicity Bioassay on SMPMs of Annonacin

**Figure 9:**
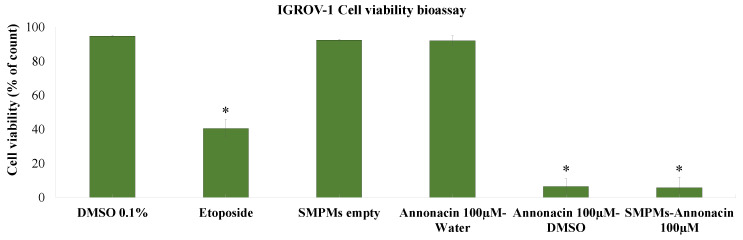
Cell viability by dye exclusion against ovarian carcinoma cells (IGROV-1). Etoposide, SMPMs empty, annonacin and SMPMs hosting annonacin were evaluated at 100 µM for 24 h. Experiments were performed in triplicate and data are expressed as mean ± SD, *n* = 3, * *p* < 0.05 vs. untreated cells (DMSO 0.1%).

The cytotoxicity of the SMPMs that hosted annonacin against IGROV-1 cells was evaluated at 100 µM for 24 h. The highest activity in aqueous media was achieved with encapsulated annonacin (Figure 9) while a solution of free annonacin in water did not show significant activity. Moreover, it is worth highlighting that the cell growth inhibition value of SMPMs was very similar to that obtained with free annonacin dissolved in DMSO and both were lower than etoposide.

The transport property of the SMPMs has an important advance in direct clinical applications. Avoiding the organic solvent employment, it is decreased possible side effects in a future in vivo treatments. These results reinforce that not only water solubility despite biorecognition has been boosted. Sugar fragments that constitute the SMPMs seems to be relevant in the cell input and this also provide a stable drug transport to the target. Even degradation of the compound could be diminished by SMPMs, because of there is no contact between annonacin and cell media in contrast with application of DMSO (miscible in water) that do not prevent side reactions.

## 3. Materials and Methods

### 3.1. General Experimental Procedures

All of the fractions obtained in the bio-guided isolation and the pure ACGs were characterized by ^1^H-NMR and ^13^C-NMR spectroscopy. ^1^H and ^13^C spectra were recorded at room temperature, using CDCl_3_ as solvent, on Agilent INOVA spectrometers at 500 and 125 MHz and 600 and 150 MHz, respectively. The residual chloroform signal was set to δ 7.25 ppm for ^1^H and to δ 77.0 ppm for ^13^C. Furthermore, ^1^H-NMR and ROESYAD experiments on the encapsulated compound were carried out at 25 °C using D_2_O as solvent on an Agilent INOVA spectrometer at 500 MHz and 600 MHz. The residual peak for water was referenced to δ 4.79 ppm. HRMS were obtained on a SYNAPT G2 mass spectrometer (Waters, Milford, MA, USA). HPLC was carried out on an HPLC chromatograph with an RI detector (Merck-Hitachi, Tokyo, Japan) and an analytical column Phenomenex^®^ Luna 10 µ Silica (2) (250 × 4.60 mm, 10 µm) (CA, USA). Silica gel 0.060–0.200, 60 Å from Acros Organics (Geel, Belgium) and Lichroprep RP 18 (40–63 μm) from Merck (Darmstadt, Germany) were used for column and vacuum column chromatography. Thin layer chromatography (TLC) was run on Silica gel 60 F_254_ and Silica gel 60 RP-18 F_254_S aluminum sheets from Merck (Darmstadt, Germany). For further purification, preparative layer chromatography (PLC) Silica gel 60 F_254_ 0.5 mm was used and this was also supplied by Merck (Darmstadt, Germany).

### 3.2. Chemicals

Ethyl acetate and *n*-hexane were purchased from VWR International (Radnor, PA, USA). Methanol and dichloromethane were obtained from Fisher Scientific (Loughborough, UK) and dimethyl sulfoxide and urea were supplied by Panreac Quimica SAU (Castellar del Vallés, Barcelona, Spain). Deuterated chloroform for spectroscopic experiments was obtained from VWR Chemicals Prolabo^®^ (Leuven, Belgium). For the cytotoxicity bioassays, Dulbecco’s Modified Eagle’s Medium (DMEM) was supplied by Lonza (Verviers, Belgium), premixed phosphate buffer saline solution (PBS, 10×) was supplied by Roche (Steinheim, Germany), fetal bovine serum, penicillin/streptomycin, l-glutamine, sodium pyruvate, trypsin and minimum essential medium non-essential amino acids (MEM NEAA), RPMI 1640 medium were purchased from Gibco (Paisley, UK). α-Cyclodextrin (α-CD) was obtained from Tokyo Chemical Industry (TCI Europe, Zwijndrecht, Belgium). Water (type I) was obtained from an Ultramatic system from Wasserlab. Lipase from porcine pancreas (L3126; enzymatic activity of 100–500 units), mucin from porcine stomach type II (M2378), pancreatin from porcine pancreas (P7545; 8 × U.S.P. specifications) and pepsin from porcine stomach mucosa (P7000; enzymatic activity of 500 units/mg solid) were obtained from Sigma Aldrich (Steinheim, Germany).

### 3.3. Plant Material

Leaves from the ‘Fino de Jete’ variety were collected in October 2014 from an experimental field located in Almuñecar (Spain) (36°44′2.1″ N 3°41′26.6″ O). Leaves, (2.2 Kg) were dried at room temperature pulverized in an industrial mill and kept at room temperature in absence humidity prior to extraction.

### 3.4. Extraction and Isolation

Dried material (2.2 Kg) was extracted with 4 L of *n*-hexane using the Soxhlet method (159 g, 7.4% yield). Defatted material was extracted with dichloromethane to yield 140 g after removal of the solvent under reduced pressure (E_1_). This residue was further purified by vacuum column chromatography with reverse phase silica gel (RP-18) with different ratios of MeOH:H_2_O from 100% water to 100% methanol with increases of 20%. Seven fractions were obtained (E_1.1_–E_1.7_). Fraction E_1.6_ (28 g, 1.3% yield) showed the lowest cell viability and it was therefore subjected to a new separation by silica gel column chromatography using a gradient of hexane:ethyl acetate (from 5 to 100% ethyl acetate with increases of 10%) as eluent. In this case, ten fractions (E_1.6.1_–E_1.6_._10_) were obtained and the most active fraction (E_1.6.5_, 2.57 g, 0.12% yield) was selected for further resolution. E_1.6.5_ was separated by column chromatography on silica gel using a gradient of hexane:ethyl acetate in the same way as described above. Thirteen fractions (E_1.6.5.1_–E_1.6.5.14_) were collected and combined on the basis of similar TLC patterns. The presence of ACGs was confirmed by NMR experiments and fractions E_1.6.5.7_–E_1.6.5.12_ were confirmed to be active in cell bioassays. Fraction E_1.6.5.7_ led to the isolation of the known ACG molvizarin (**1**) by HPLC with an analytical Phenomenex^®^ Luna 10 µ Silica (2) column, with hexane:ethyl acetate:acetone (7:2:1) as solvent at flow rate of 1 mL/min, t_R_ 40.6 min. Purification and evaluation of the remaining fractions (E_1.6.5.8_–E_1.6.5.10_) by CC on silica gel (with a gradient of hexane:ethyl acetate) and PLC (using as the organic phase a mixture of CHCl_3_:MeOH:acetone:H_2_O (13:7:1:3) as eluent) led to the isolation of cherimolin-1 (**2**), motrilin (**3**), annonacin (**4**) and annonisin (**5**).

ACGs **1**–**4** have been reported previously in seeds from the species studied here [30,31,32]. Nevertheless, all of the compounds are described for the first time in *Annona cherimola* Mill. deciduous leaves. Annonisin (**5**) has been isolated from *Annona atemoya* seeds [33] and it is reported for the first time in this species.

### 3.5. Synthesis of SMPMs-ACGs

A variation of the method of Mejías and co-workers [28] was used. 40 mg of annonacin was dissolved in 4 mL of THF (0.0168 mM). 160 mg of α-CD (0.164 mmol) and 160 mg of urea (2.66 mmol) were weighed together and dissolved in 4 mL of water type I with sonication to enhance the dissolution process. Annonacin solution was added dropwise to the cyclodextrin solution and this mixture was stirred for 24 h at 40 °C followed by dialysis for 0.5 h. A Spectra/Pore^®^ membrane (Sigma Adrich, Steinheim, Germany) with 6000–8000 AMU cut off was loaded with the solution and the sample was lyophilized to obtain a fine powdery white solid.

### 3.6. Electron Microscopy

The morphology and particle size of SMPMs-ACGs were studied using a JEOL2100 transmission electron microscope (JEOL, Tokyo, Japan). An LC300-Cu-150 grid with lacey carbon film was employed to prepare all the samples. Each electron microscopy sample was prepared by dispersing the solid in a miliQ quality degree water and dropping the solution onto a copper grid with lacey carbon. The copper grid with the sample was dried overnight to evaporate the solvent before microscopic analysis. The staining process was avoided to facilitate the correct elucidation of the micelle structures.

### 3.7. Encapsulation Efficiency

The percentage of annonacin inside the supramolecular polymer micelles was determined by breaking the shell to release the bioactive compound. The samples were dissolved in CHCl_3_ to remove this external layer of the core/shell systems and it was vortexed once. The organic solvent released the core compound and, subsequently, the absorbance of annonacin was measured by UV-Vis spectroscopy (Varian Cary 50 BIO spectrophotometer, Spijkenisse, The Netherlands). Absorbance values were converted to concentration values with a calibration curve (Figure S7).

### 3.8. Compound Release

A 1000 ppm concentration of the encapsulated system was analyzed by UV-Vis spectroscopy (Varian Cary 50 BIO spectrophotometer). Successive spectra were acquired at different time intervals and the variation in the absorbance bands for annonacin (**4**) was studied. Scans from 200 to 600 nm were recorded every 30 s for the first 10 min, then a spectrum was acquired every minute up to 30 min and thereafter a spectrum every 15 min up to 24 h. Each experiment was carried out at 36 °C in saline buffer at pH 7.4, in the absence of light and with stirring, to simulate the conditions of a living organism.

### 3.9. Bioavailability Studies

The bioavailability of **4** when encapsulated in SMPMs of α-CD and urea was studied in a simulated gastrointestinal model based on the method described by Mejias et al. [28]. For the sake of comparison, this assay was also carried out on free annonacin. The concentration to calculate bioavailability was obtained from the calibration curve mentioned previously, with the absorbance values measured at 284.9 nm. The bioavailability index was determined using the following expression [53]:

Bioavailability (%) = 100 × (Cm/Ci)

where Cm is the concentration of annonacin solubilized in the supernatant and Ci is the concentration of the initial amount added to the simulated digestive system.

### 3.10. Cell Lines and Cell Cultures

IGROV-1 (human ovarian carcinoma), HeLa (human cervix carcinoma), CHO (Chinese hamster ovary) and HEK-293 (human embryonic kidney 293) cells were cultured as monolayers in DMEM (GIBCO) supplemented with 10% fetal bovine serum, 5% glutamine, 5% non-essential amino acids, 5% penicillin-streptomycin and 5% sodium pyruvate. Cells were maintained in a HERA Cell 150i (Thermo Scientific) incubator at 37 °C, 5% CO_2_ and 95% humidity.

### 3.11. Cell Viability Assay

Tumoral cell lines (HeLa and IGROV-1) and non-tumoral cells (HEK-293) were used for the experiments. 1.5 × 10^5^ cells per mL were seeded in 6-well plates (VWR, langenfeld, Germany) in complete medium. Extracts and pure compounds at 100 ppm and 100 µM, respectively, were dissolved in DMSO (0.1% *v*/*v*) and added for 24 h. Free annonacin in DMSO was also evaluated in the concentration range 6.25 to 100 µM for 24 h. Further studies with supramolecular polymer micelles of annonacin diluted in PBS at 100 µM (170.5 ppm), as well as SMPMs empty at 170.5 ppm, were performed for 24 h. SMPMs empty has been obtained following the same synthetic procedure for SMPMs-ACGs, but without adding annonacin. The amount required for the bioassay was calculated taking into account the encapsulation efficiency to afford the polymeric micelle (35%) to a final concentration of 100 µM of annonacin. Control cultures, including cells treated either with 0.1% DMSO or PBS 1X, were also included in each experiment. Cell viability was evaluated by the Trypan blue assay. Trypan blue solution (0.4% Sigma Aldrich, Steinheim, Germany) was mixed 1:1 with a sample of control or treated cells. After incubation for 2 min, a fraction of blue-stained cells was assessed using an Automated TC20 Cell Counter (Bio-Rad). IC_50_ values were determined with GraphPad (Prism software v. 5.00). Experiments were carried out at least in triplicate and data are expressed as the mean ± SD. All data were analyzed using one-way ANOVA and values were considered to be statistically significant when *p* < 0.05.

### 3.12. Fluorescence Microscopy

HeLa cells were grown on 11 × 22 mm coverslips (Thomas Scientific, Swedesboro, NJ, USA) and incubated for 20 h. with annonacin at 100 µM in culture medium as described above. Caspase-3 activity assay was performed according to the manufacturer’s protocol [41,54]. Primary rabbit active caspase-3 antibody (1:500 dilution) and secondary fluorescein isothiocyanate (FITC)-conjugated goat anti-rabbit IgG antibody (dilution 1:100) were used for the incubation. The samples were observed using a Zeiss Axiophot microscope (Carl Zeiss, Oberkochen, West Germany) and images were taken with a Zeiss Axiocam 503 camera (Carl Zeiss, Jena, Germany).

Fluorescence intensity measurements, *n* = 3, and multiple ROIs (regions of interest) were measured from groups of 20–30 cells. Images were transformed to 8-bit gray scale and fluorescence intensity was analyzed with ImageJ 1.53 e, using the particle analysis function. One-way ANOVA followed by a Tukey multiple comparisons test was performed to compare mean fluorescence intensity between the treatment groups using the GraphPad Prism version 5.0 (GraphPad Software, San Diego, CA, USA). Triplicate measurements were done in 3 randomly selected areas of each of the cell culture fields with a background correction. All data were expressed as the mean ± SEM.

## 4. Conclusions

The present study shows the isolation of five ACGs that are described for the first time in *A. cherimola* deciduous leaves. One of the ACGs, annonisin (**5**), is reported for the first time in this species. In vitro cytotoxicity experiments showed strong toxicity against HeLa and IGROV-1 tumoral cell lines. Specially, annonacin (**4**) was found to be four- and two-times more potent in tumoral cells than in non-tumoral human embryonic kidney cells (HEK-293) at 50 µM. In view of these results, compound 4 was encapsulated in an effort to improve solubility and enable the development of nutraceutical or food supplements. SMPMs were successfully synthesized by employing α-cyclodextrin and urea as building blocks, as evidenced by TEM images and NMR studies of cyclodextrin signals. The structure corresponded to a core/shell system with a wall thickness close to 8 nm. According to molecular models developed with semi-empirical methods, between six and eight cyclodextrin units form the shell structure. Studies on the delivery of 4 at 37 °C in PBS showed a release process that began after 17 h of being dissolved when it was encapsulated in the supramolecular polymer micelles. Furthermore, compound 4 remained stable in solution after being delivered. Kinetic analysis fitted with the Hixson–Crowell model is consistent with a release procedure by size reduction but with the geometrical form kept constant. Bioavailability studies showed an increase in stability and uptake capability of 4 when it was encapsulated in the SMPMs. These results highlight the use of supramolecular polymer micelles as nanocarriers for annonaceous acetogenins due to the improvements in the solubility, bioavailability and activity of annonacin. This by-product could be a starting point for the development of nutraceuticals with anticancer properties. These findings suggest that SMPMs of annonacin may become a promising candidate for clinical antitumor therapy and reduced side effects. Further in vivo experiments are needed.

## 5. Patents

The work reported in this manuscript has resulted in the filing of a patent whose identifier is: P201900173.

## Supplementary Materials

The following are available online, Physical data. ^1^H and ^13^C spectra. Figure S1: Cell viability by dye exclusion against ovarian carcinoma cells (IGROV-1) for the first (A), second (B) and third (C) fractionations of *A. cherimola* deciduous leaves. Extracts were evaluated at 100 ppm for 24 h. Experiments were performed in triplicate and data are expressed as mean ± SD, *n* = 3. Figure S2: D_2_O ^1^H-NMR comparison between α-CD, α-CD/urea polyrotaxane, SMPMs-ACGs and SMPMs-ACGs with phenol, from bottom to top. Figure S3: ROESYAD 2D experiments. Figure S4: SMPMs wall thickness size distribution. Figure S5: SMPMs size distribution. Figure S6: PM3 model of the SMPM shell in the case of two units of α-CD and thirty-two molecules of urea. (A) Biased geometry, with a distance of 26.46 Å between cyclodextrin molecules. (B) Truncated cone geometry, with a distance of 17.47 Å between cyclodextrin molecules (hydrogens are not represented). Figure S7: Calibration curve for annonacin in CHCl_3._
