# Supplementary material for: Bio-Guided Isolation of Acetogenins from Annona cherimola Deciduous Leaves: Production of Nanocarriers to Boost the Bioavailability Properties
Source: Molecules. 2020 Oct 21;25(20):4861. doi: 10.3390/molecules25204861 (PMC7587976; doi:10.3390/molecules25204861)

molecules

MDPI

Supporting information

Bio-guided Isolation of Acetogenins from *Annona cherimola* deciduous leaves: Production of Nanocarriers to Boost the Bioavailability Properties.

M. Teresa Gutiérrez [1†], Alexandra G. Durán [1†], Francisco J. R. Mejías[1], José M.G. Molinillo[1], Diego Megias[2], Manuel M. Valdivia[3] and Francisco A. Macías[1*]

[1] Allelopathy Group, Department of Organic Chemistry, Institute of Biomolecules (INBIO), Campus de Excelencia Internacional (ceiA3), School of Science, University of Cadiz, 11510 Puerto Real, Cadiz, Spain; mariateresa.gutierrezva@alum.uca.es (M.T.G.); alexandra.garcia@uca.es (A.G.D.); javi.rodriguezmejias@uca.es (F.J.R.M.); chema.gonzalez@uca.es (J.M.G.M.); famacias@gm.uca.es.(F.A.M.)

[2] Confocal Microscopy Unit, Spanish National Cancer Research Center (CNIO), Madrid, E-28029, Spain; dmegias@cnio.es (D.M.)

[3] Department of Biomedicine, Biotechnology and Public Health, School of Science, Institute of Biomolecules (INBIO), University of Cadiz, 11510 Puerto Real, Cádiz, Spain; manuel.valdivia@uca.es (M.M.V.)

* Correspondence: famacias@gm.uca.es. Telephone: +34-956-012770

† These authors contributed equally to this work

molecules

Index

Physical data S3

^{1}H and ^{13}C spectra S4–S8

Figure S1. Cell viability by dye exclusion against ovarian carcinoma cells (IGROV-1) for the first (**A**), second (**B**) and third (**C**) fractionations of *A. cherimola* deciduous leaves. Extracts were evaluated at 100 ppm for 24 h. Experiments were performed in triplicate and data are expressed as mean ± SD, n = 3. S9

Figure S2. D_2O ^{1}H NMR comparison between α-CD, α-CD/urea polyrotaxane, SMPMs-ACGs and SMPMs-ACGs with phenol, from bottom to top S10

Figure S3. ROESYAD 2D experiments S11

Figure S4. SMPMs wall thickness size distribution S12

Figure S5. SMPMs size distribution S13

Figure S6. PM3 model of the SMPM shell in the case of two units of α-CD and thirty-two molecules of urea. (A) Biased geometry, with a distance of 26.46 Å between cyclodextrin molecules. (B) Truncated cone geometry, with a distance of 17.47 Å between cyclodextrin molecules (hydrogens are not represented) S14

Figure S7. Calibration curve for annonacin in $CHCl_3$ S15

Figure S8. Critical micellar concentration experiment S16

Figure S9. XRD experiment of (A) α-cyclodextrin and (B) SMPMs-ACGs S17

molecules

MDPI

Physical data

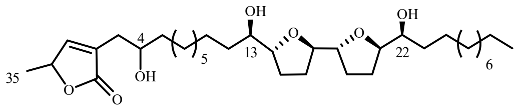

Molvizarin (**1**)

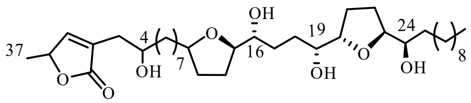

Cherimolin-1 (**2**)

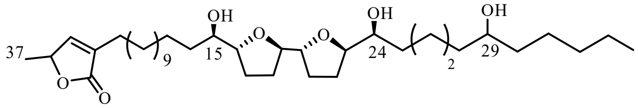

Motrilin (**3**)

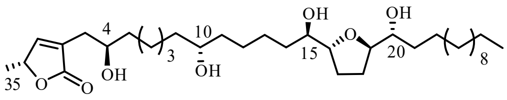

Annonacin (**4**)

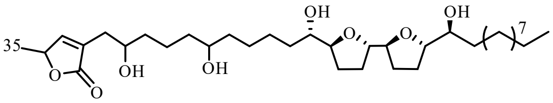

Annonisin (**5**)

Molvizarin (**1**). This compound was obtained as a colourless oil in 0.001% yield; HRMS (ESI) calcd for $C_{35}H_{61}O_7$ $[M-H]^-$: 593.4417, found: 593.4460.

Cherimolin-1 (**2**). This compound was obtained as a colourless oil in 0.0002% yield; HRMS (ESI) calcd for $C_{37}H_{65}O_8$ $[M-H]^-$: 637.4679, found: 637.4723.

Motrilin (**3**). This compound was obtained as a colourless oil in 0.0002% yield;

HRMS (ESI) calcd for $C_{37}H_{65}O_7$ $[M-H]^-$: 621.4679, found: 621.4717.

Annonacin (**4**). This compound was obtained as a yellow oil in 0.003% yield; HRMS (ESI) calcd for $C_{35}H_{64}O_7$ $[M+H]^+$: 597.4736, found: 597.4730.

Annonisin (**5**). This compound was obtained as a colourless oil in 0.001% yield; HRMS (ESI) calcd for $C_{36}H_{63}O_{10}$ $[M+HCOO]^-$: 655.4421, found: 655.4424.

molecules
MDPI

^{1}H-NMR and ^{13}C-NMR Spectroscopy of ACGs 1–5 (^{1}H NMR 600 MHz, ^{13}C NMR 150 MHz, $CDCl_3$)

Molvizarin (**1**)

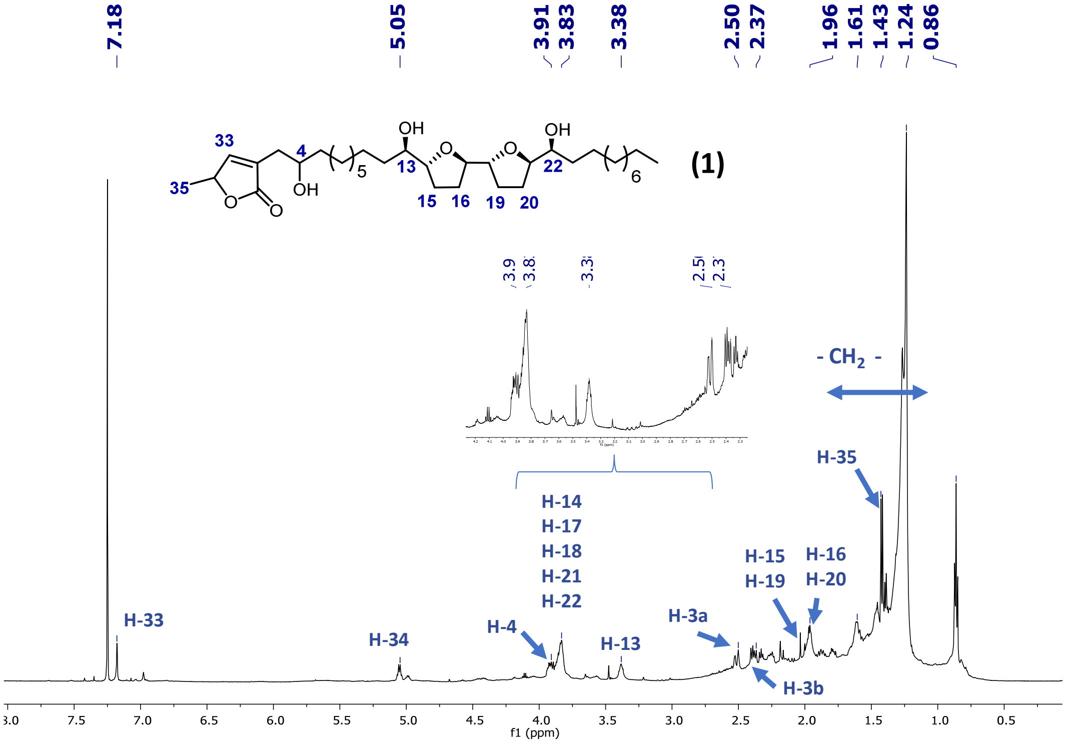

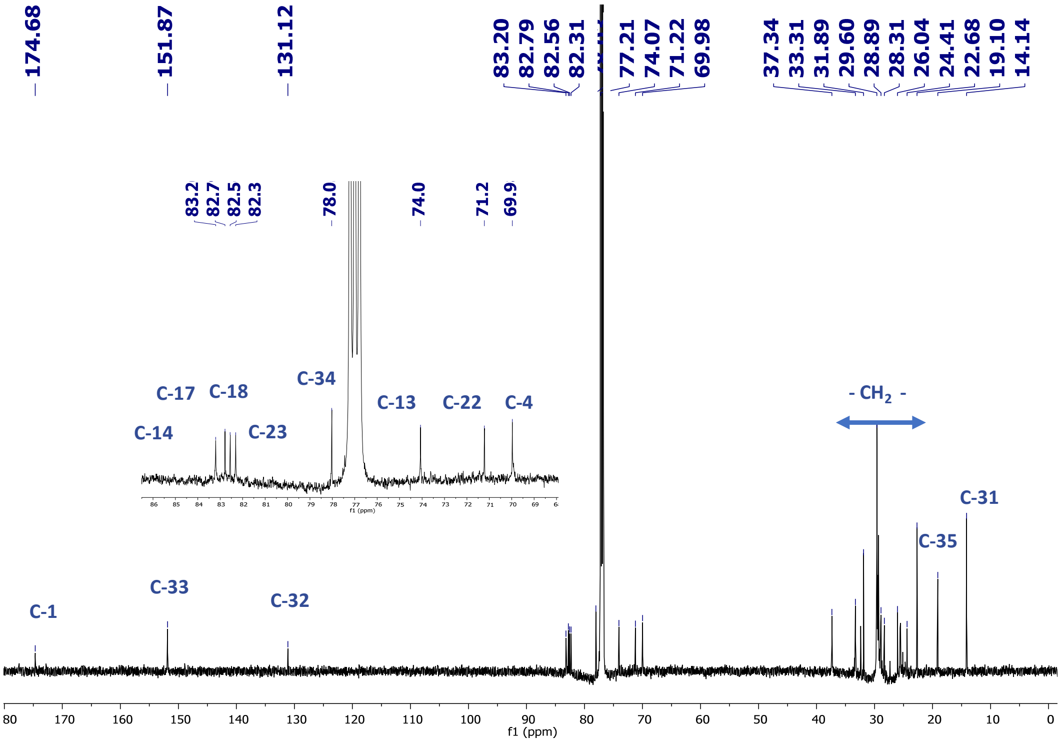

Cherimolin-1 (**2**)

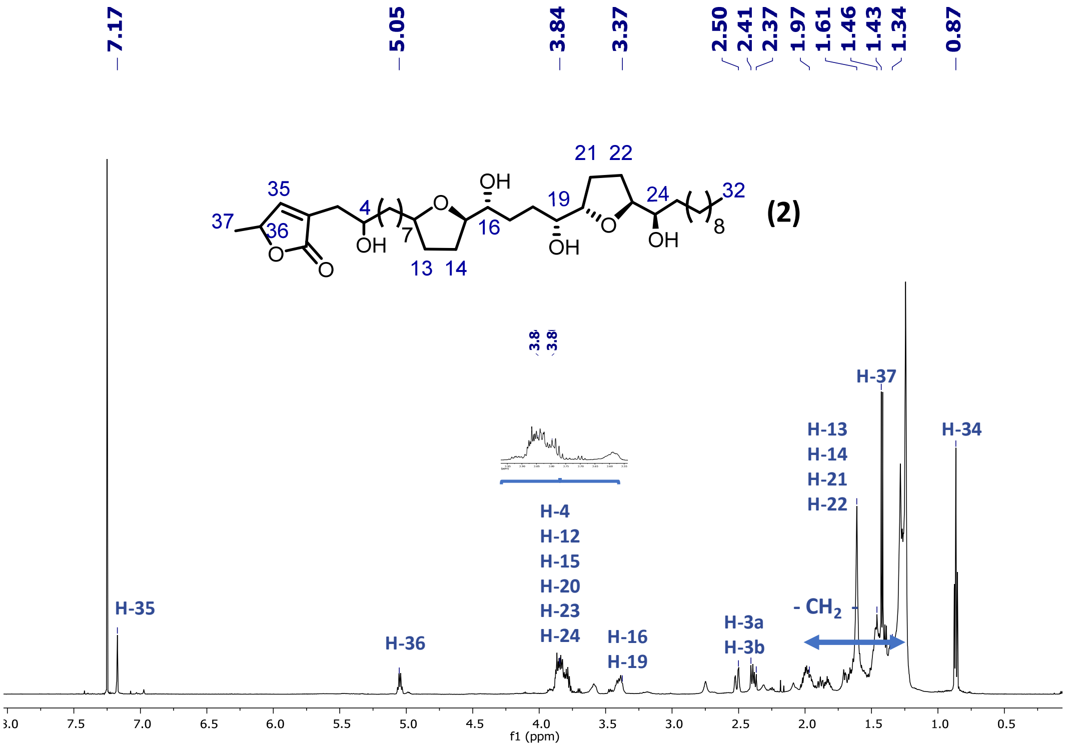

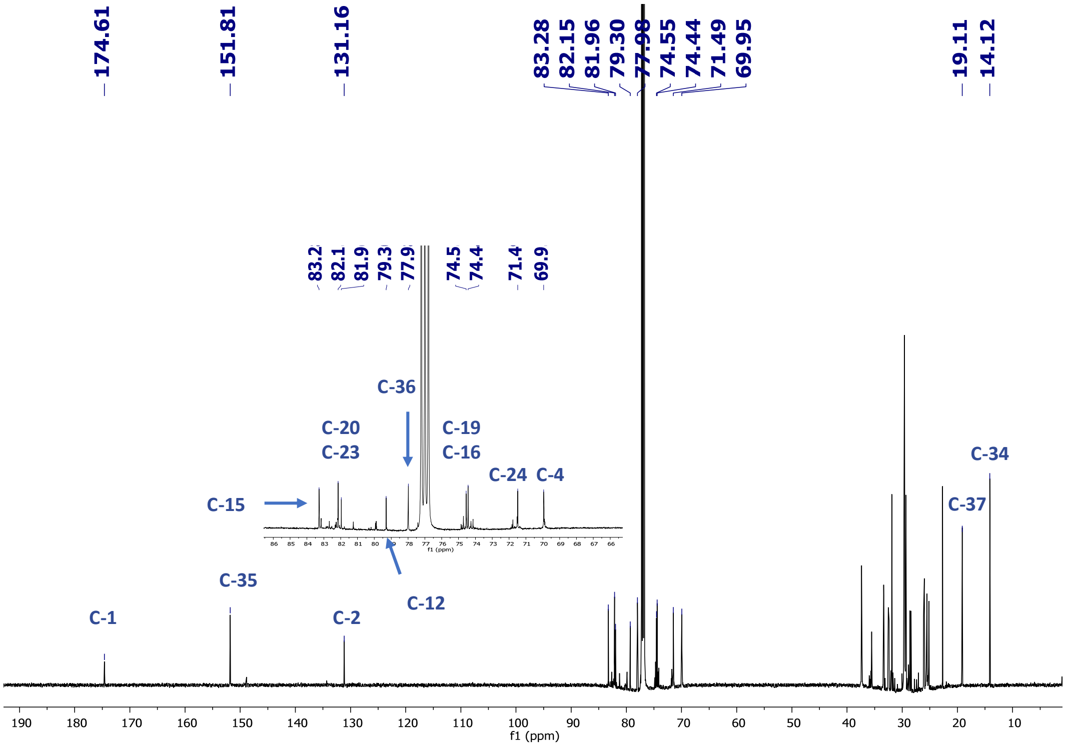

molecules
MDPI

Motrilin (**3**)

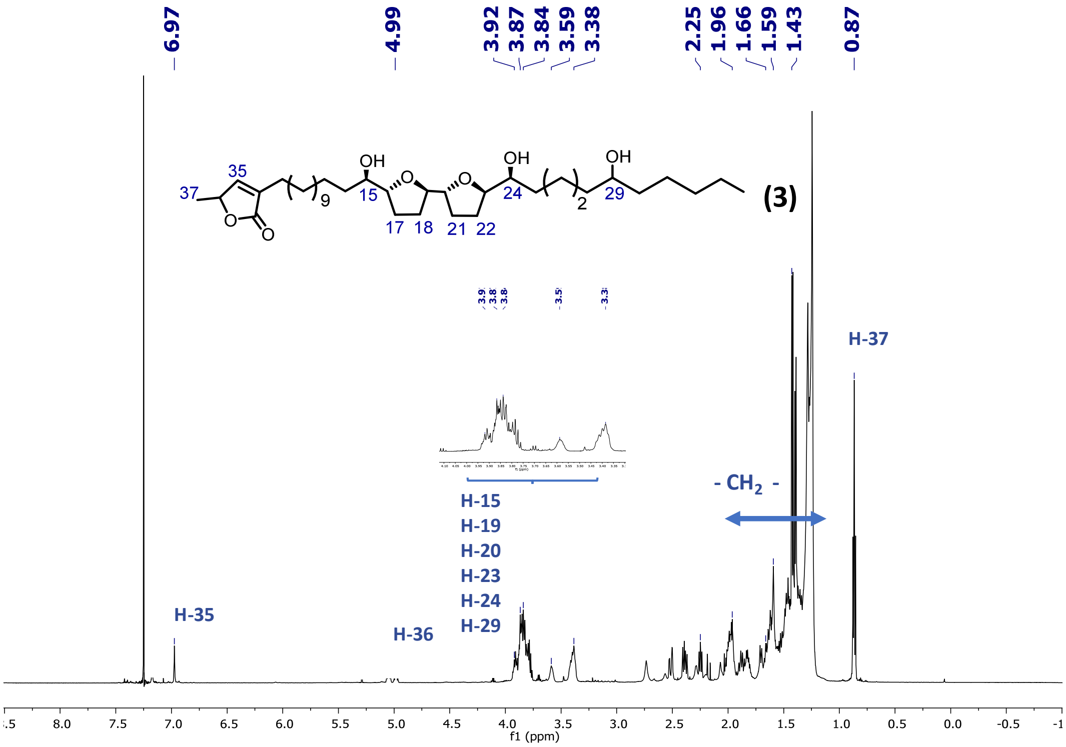

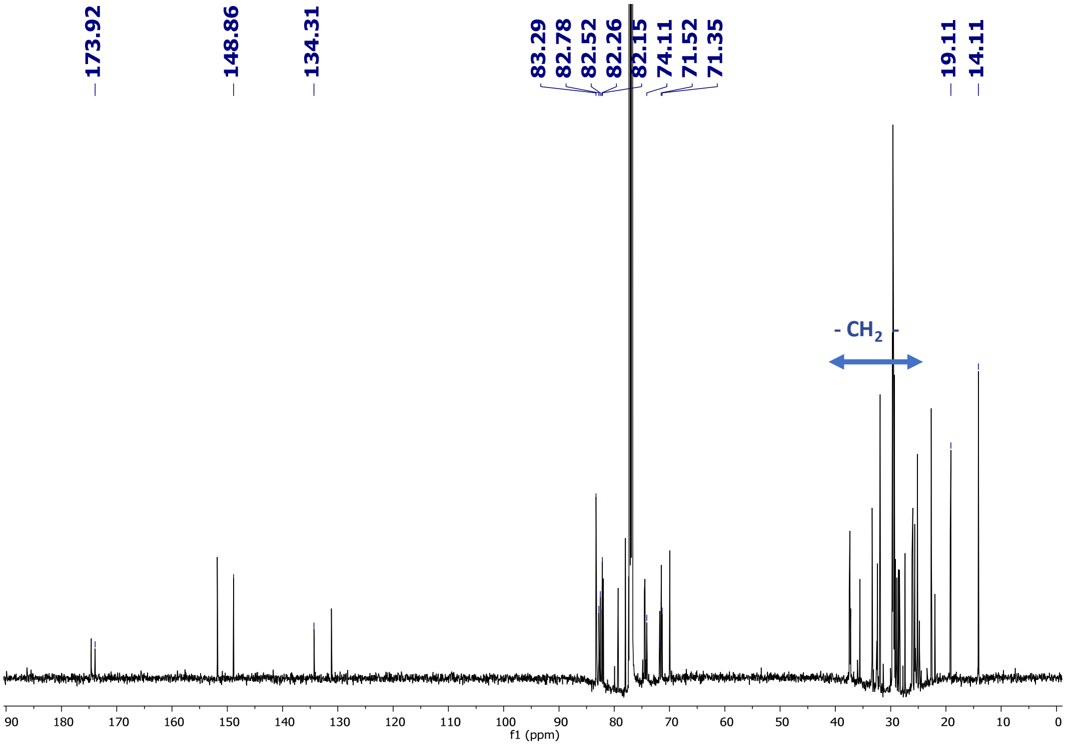

Annonacin (**4**)

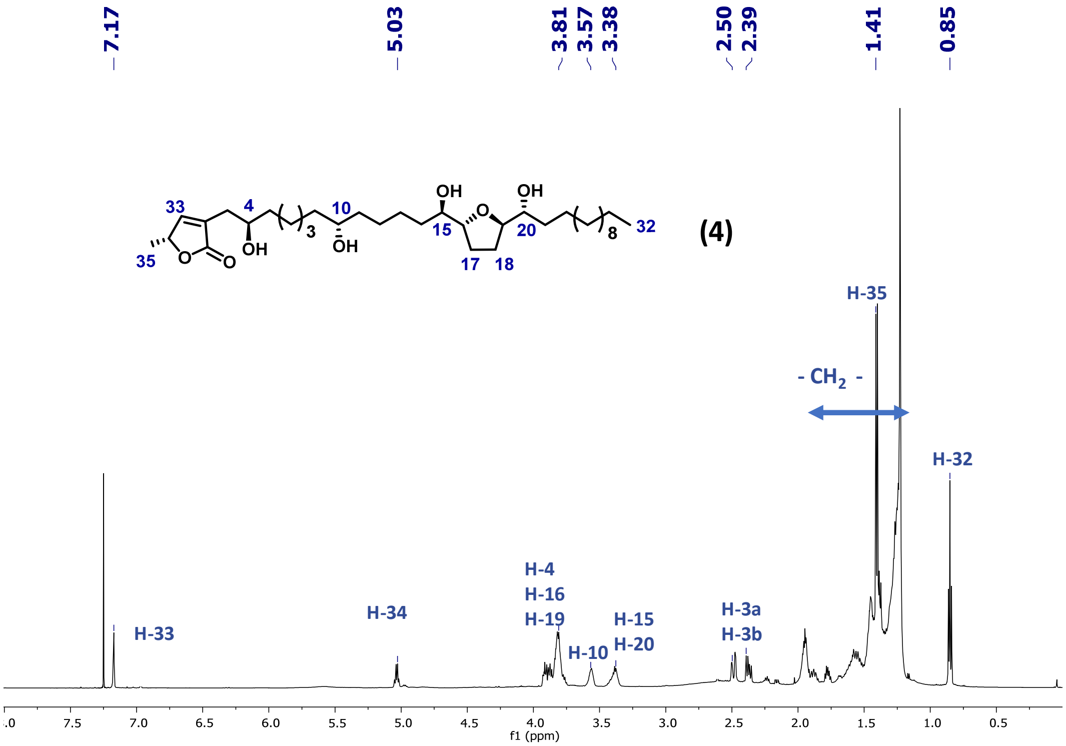
7.17
5.03
3.81
3.57
3.38
2.50
2.39
1.41
0.85
(4)
H-35
- CH2 -
H-32
H-4
H-16
H-19
H-34
H-33
H-15
H-20
H-10
H-3a
H-3b
f1 (ppm)

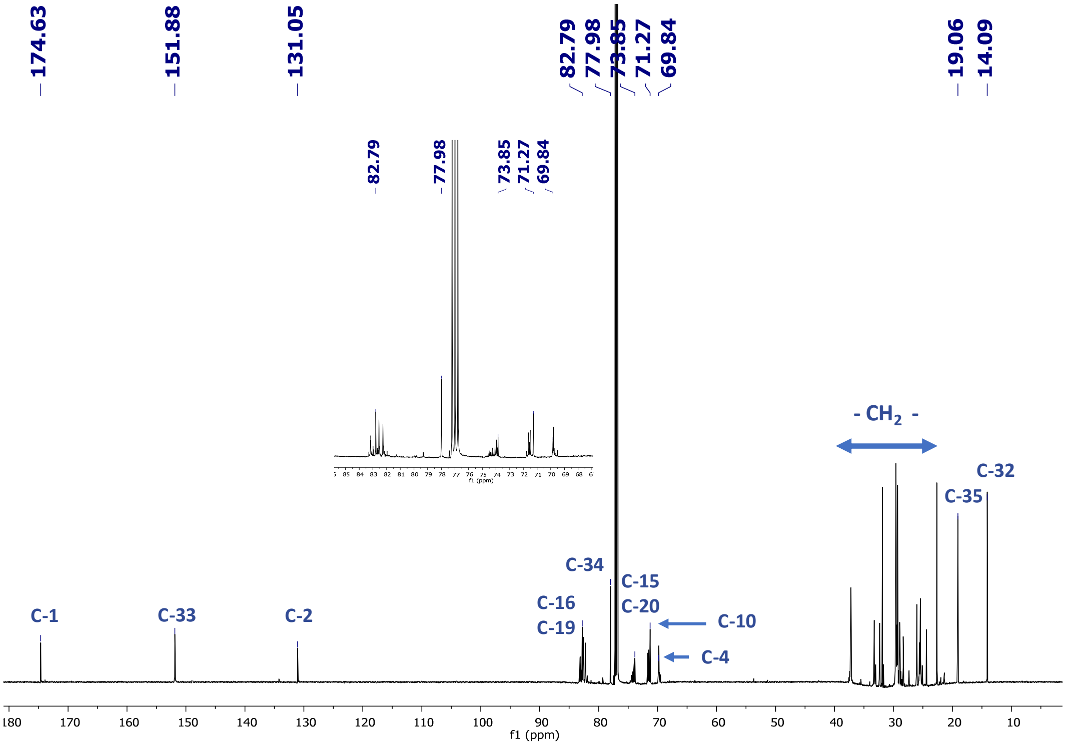
174.63
151.88
131.05
82.79
77.98
73.85
71.27
69.84
19.06
14.09
- CH2 -
C-32
C-35
C-34
C-15
C-20
C-16
C-19
C-10
C-4
C-1
C-33
C-2
f1 (ppm)

Annonisin (**5**)

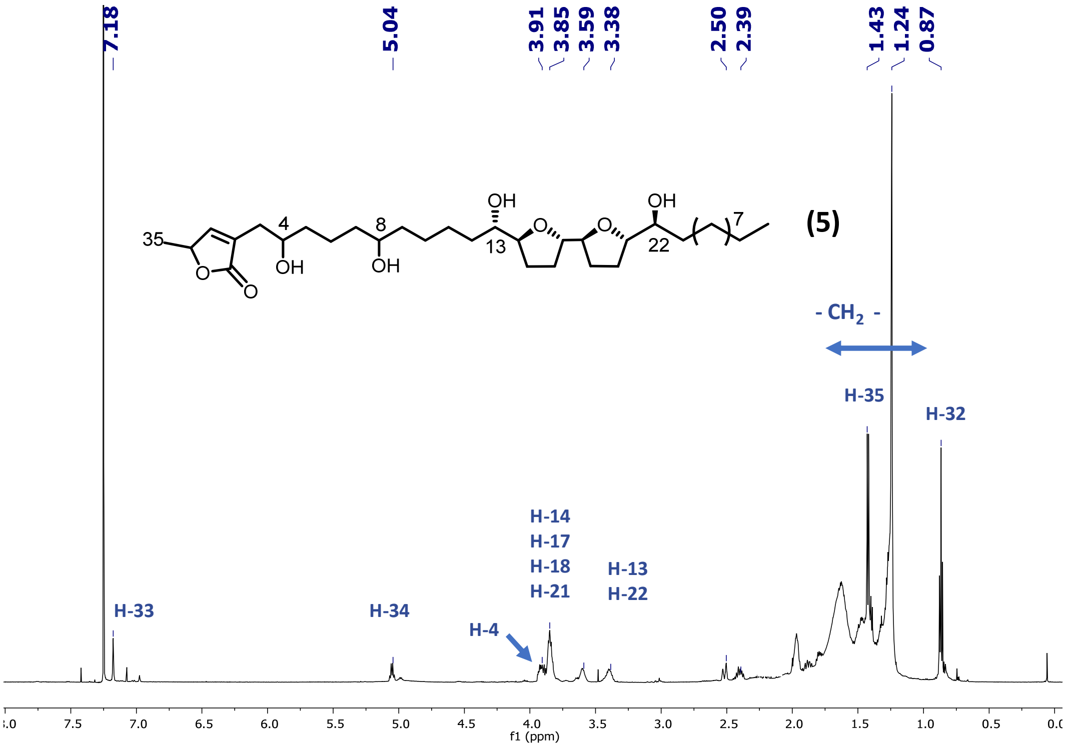
7.18
5.04
3.91
3.85
3.59
3.38
2.50
2.39
1.43
1.24
0.87
(5)
- CH2 -
H-35
H-32
H-14
H-17
H-18
H-21
H-13
H-22
H-33
H-34
H-4
f1 (ppm)

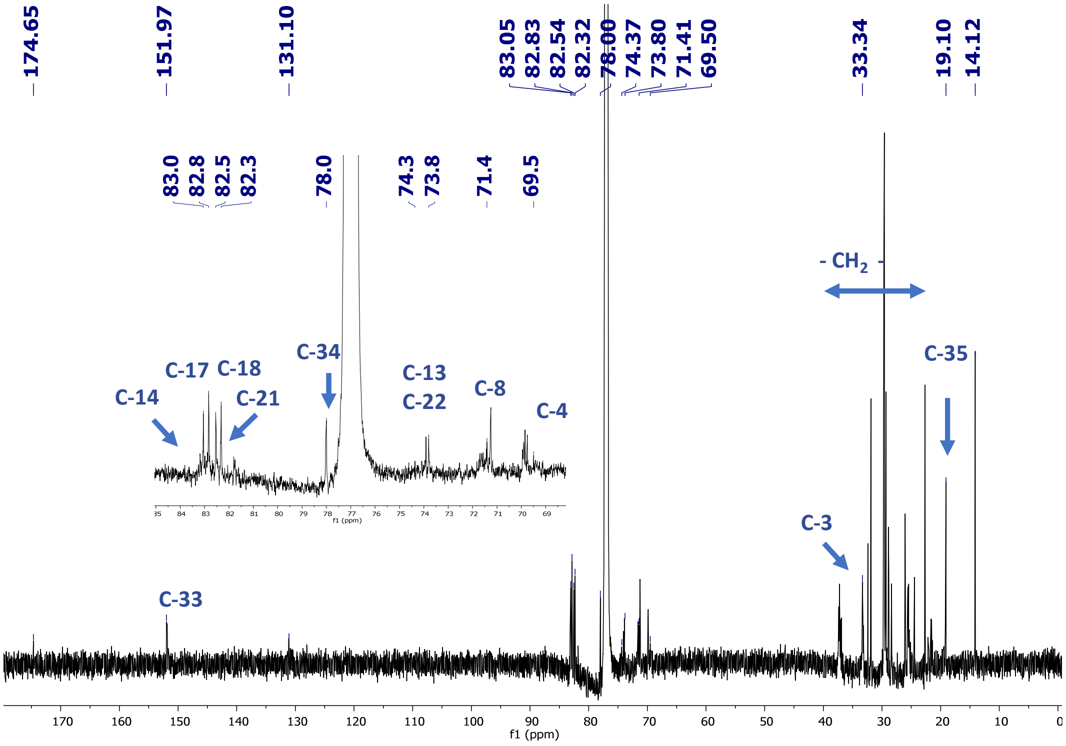
174.65
151.97
131.10
83.05
82.83
82.54
82.32
78.00
74.37
73.80
71.41
69.50
33.34
19.10
14.12
83.0
82.8
82.5
82.3
78.0
74.3
73.8
71.4
69.5
- CH2 -
C-17
C-18
C-14
C-21
C-34
C-13
C-22
C-8
C-4
C-35
C-3
C-33
f1 (ppm)

molecules

MDPI

Figure S1. Cell viability by dye exclusion against ovarian carcinoma cells (IGROV-1) for the first (**A**), second (**B**) and third (**C**) fractionations of *A. cherimola* deciduous leaves. Extracts were evaluated at 100 ppm for 24 h. Experiments were performed in triplicate and data are expressed as mean ± SD, n = 3, * $p < 0.05$ vs untreated cells (DMSO 0.1%).

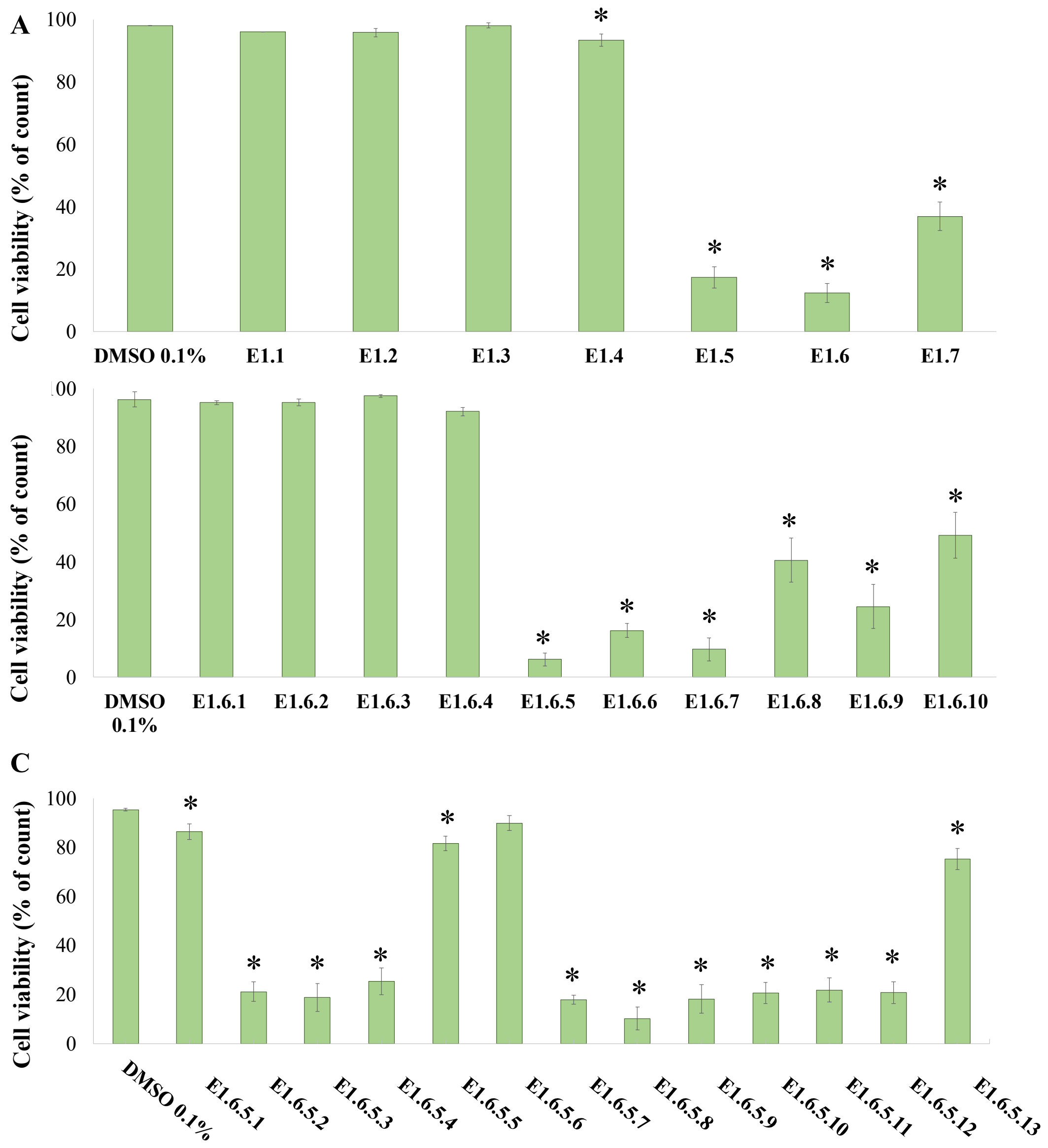

molecules

MDPI

Figure S2. D_2O ^1H NMR comparison between α-CD, α-CD/urea polyrotaxane, SMPMs-ACGs and SMPMs-ACGs with phenol, from bottom to top.

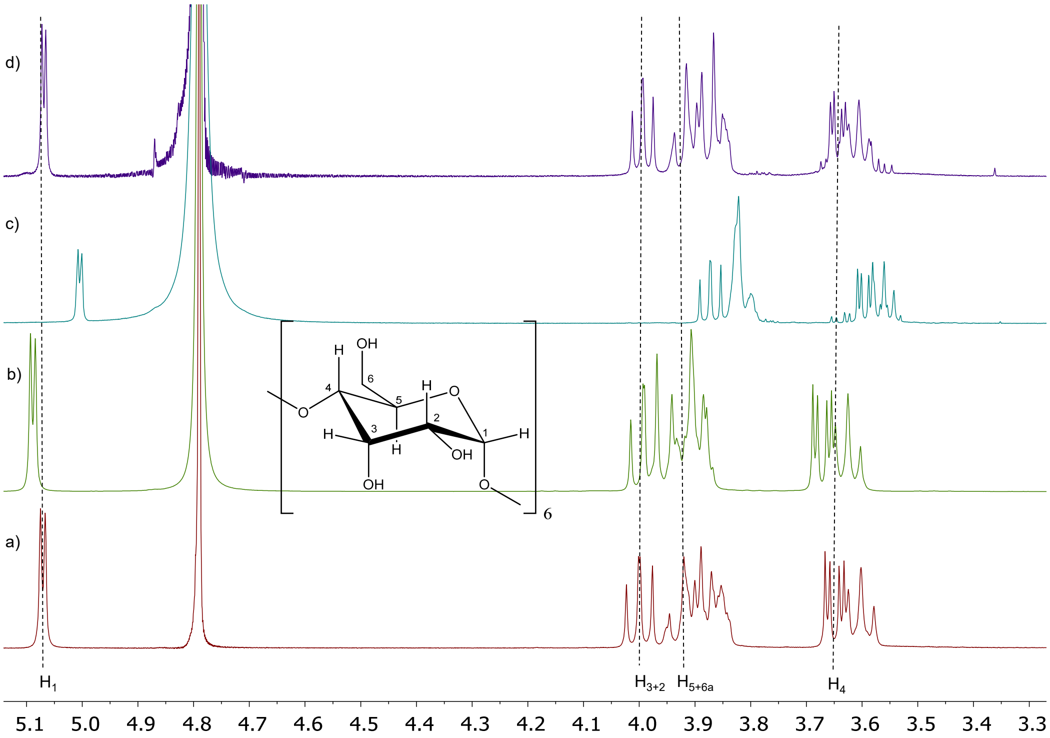

molecules

Figure S3. (Top) ^{1}H-NMR of SMPMs-ACGs/α-CD at different ppm to differenciate alpha-cyclodextrin signals and annonacin signals. (Bottom) ROESYAD 2D experiment of SMPMs-ACGs/α-CD.

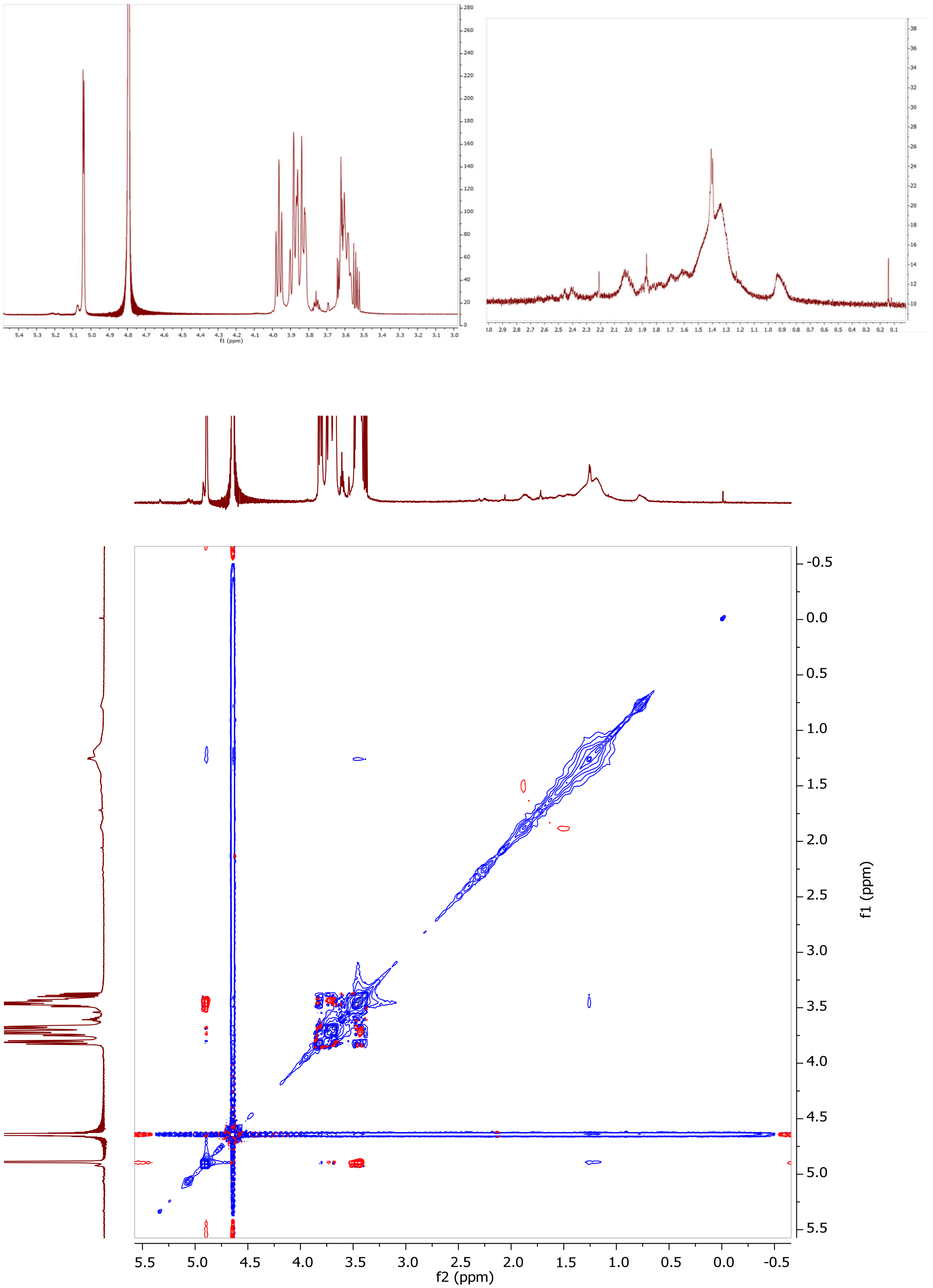

molecules

Figure S4. SMPMs wall thickness size distribution that are associated with α-CD/urea, with a mean size of 96.02 nm.

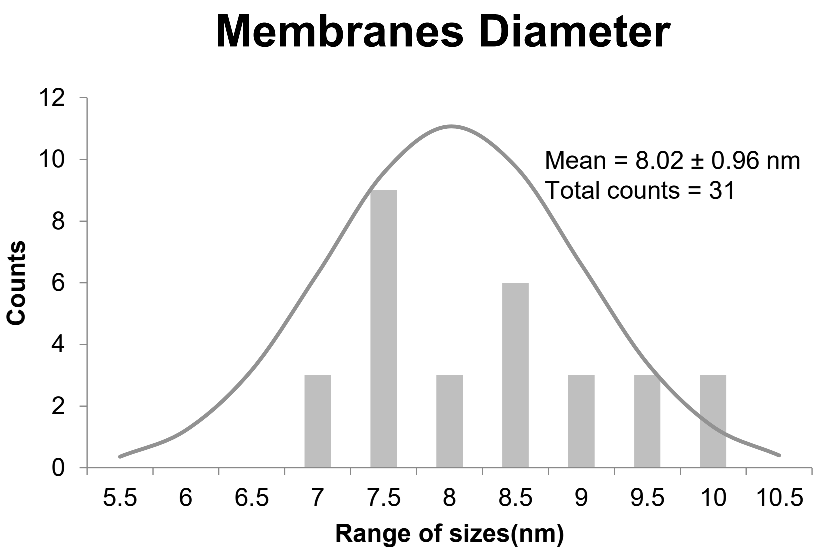

molecules

Figure S5. SMPMs size distribution that are associated with α-CD/urea, with a mean size of 96.02 nm. Two normal distributions can be observed at 60 and 140 nm of mean.

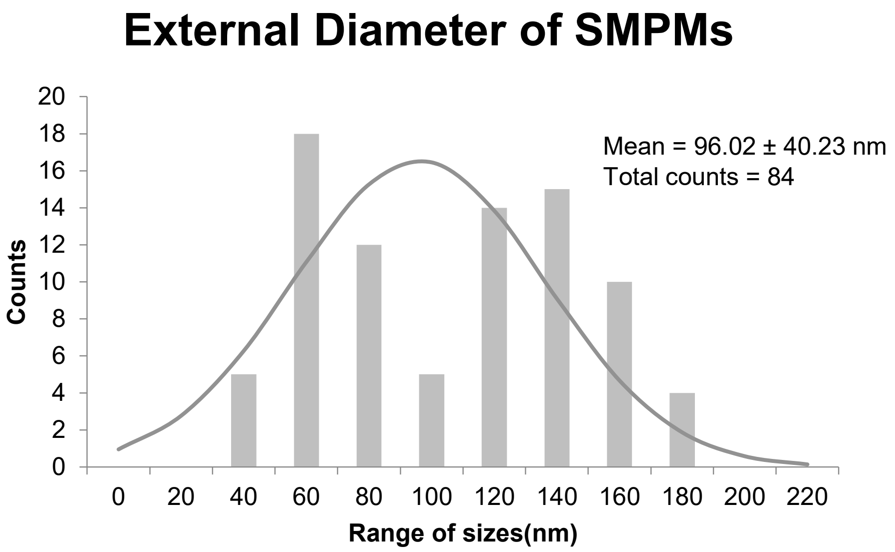

molecules

MDPI

Figure S6. PM3 model of the SMPM shell in the case of two units of α-CD and thirty-two molecules of urea. (A) Biased geometry, with a distance of 26.46 Å between cyclodextrin molecules. (B) Truncated cone geometry, with a distance of 17.47 Å between cyclodextrin molecules (hydrogens are not represented).

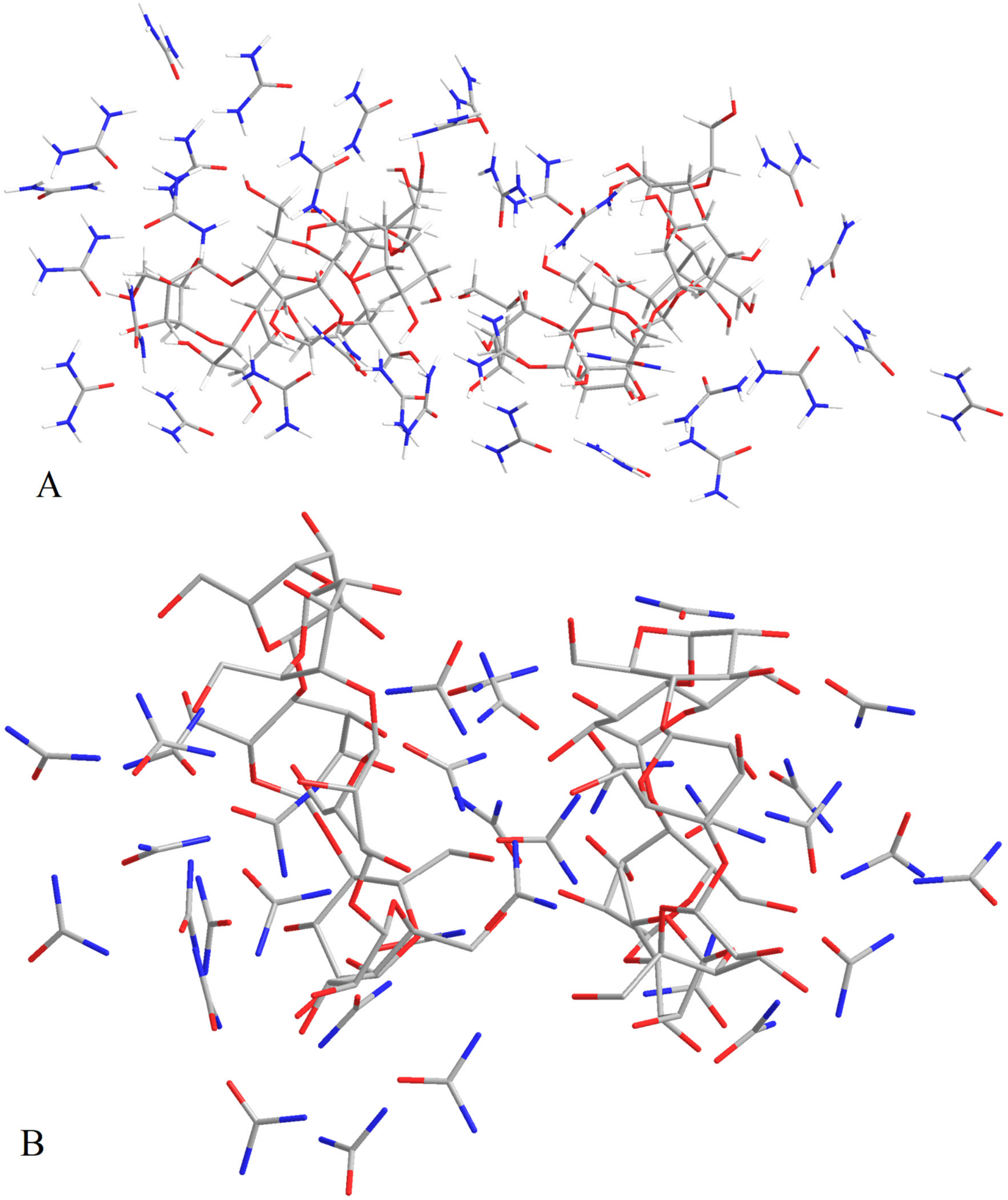

molecules

Figure S7. Calibration curve for annonacin in $CHCl_3$.

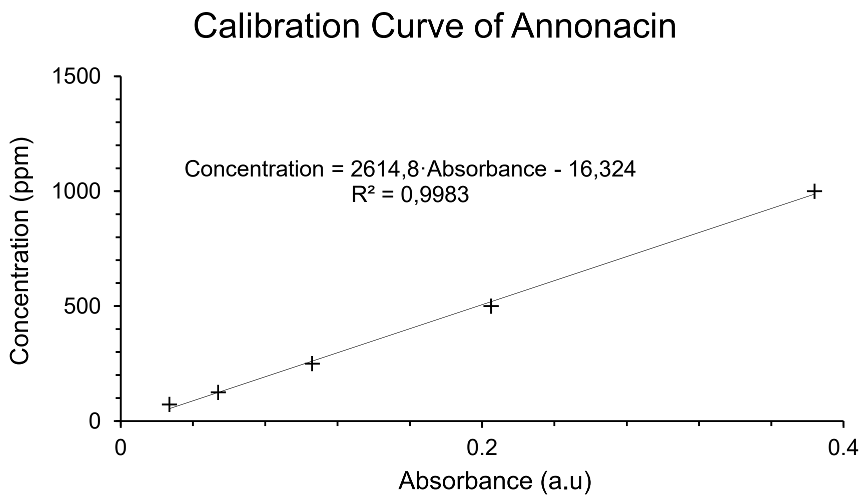

molecules

MDPI

Figure S8. Critical micellar concentration experiment

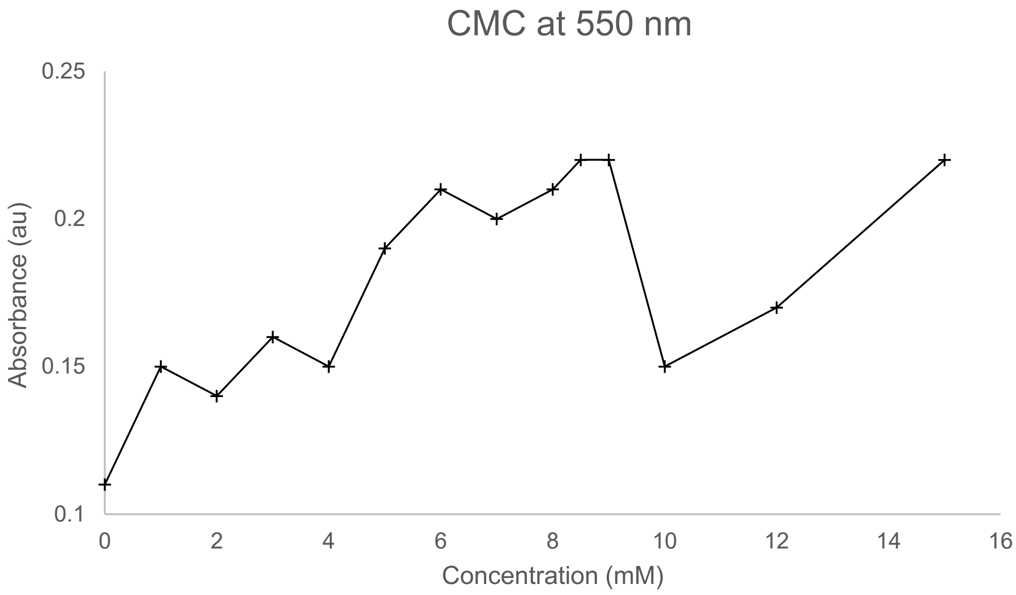

molecules

MDPI

Figure S9. XRD experiment of (A) a-cyclodextrin and (B) SMPMs-ACGs

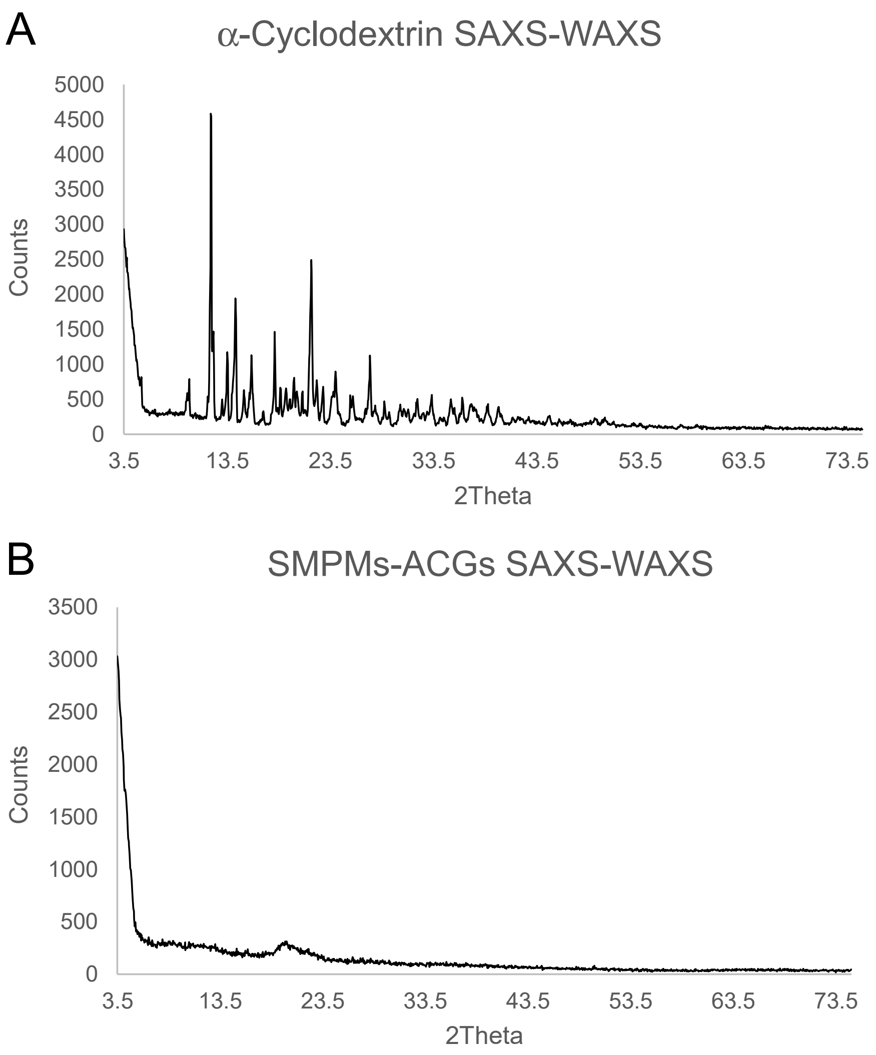

Supplement: Supplementary file 1 [file molecules-25-04861-s001.pdf]
